# Cysteine Mutagenesis of a Group II Intron-Encoded Protein Supports Splicing, Mobility, and Site-Specific Labeling

**DOI:** 10.1021/acs.biochem.5c00382

**Published:** 2025-08-29

**Authors:** Jasmine A. Harper, Sarah A. Starcovic, Neil Billington, Aaron R. Robart

**Affiliations:** Department of Biochemistry and Molecular Medicine, 5631 West Virginia University, Morgantown, West Virginia 26506, United States

## Abstract

Group II introns are self-splicing ribozymes that excise themselves from precursor RNA and integrate into new DNA locations through retromobility. Splicing is facilitated by an intron-encoded protein (IEP), a multidomain reverse transcriptase that enhances ribozyme activity and promotes formation of lariat intron–IEP ribonucleoprotein (RNP) complexes. In this study, we examined the role of conserved cysteine residues in the IEP of the group IIC intron *Ta.it.*I1 from the thermophile *Thermoanaerobacter italicus* by generating cysteine-to-methionine mutants. All variants retained near wild-type splicing efficiency, indicating that cysteine substitution does not impair maturase function. A mutation in the thumb domain significantly enhanced reverse transcription (RT) activity, whereas substitutions flanking the YADD catalytic motif led to reduced activity. Despite these variable RT effects, all mutants retained the ability to complete both steps of forward intron self-splicing and subsequently perform reverse splicing into DNA targets. Complete removal of native cysteines enabled site-specific fluorescent labeling of the IEP using maleimide–thiol chemistry without disrupting splicing or retromobility. Labeled IEPs retained activity and were successfully used to monitor RNA binding and RNP assembly under native conditions. These findings highlight the structural flexibility of IEP–intron interactions and demonstrate that site-specific IEP labeling enables real-time visualization of RNP assembly and dynamics.

## Graphical abstract

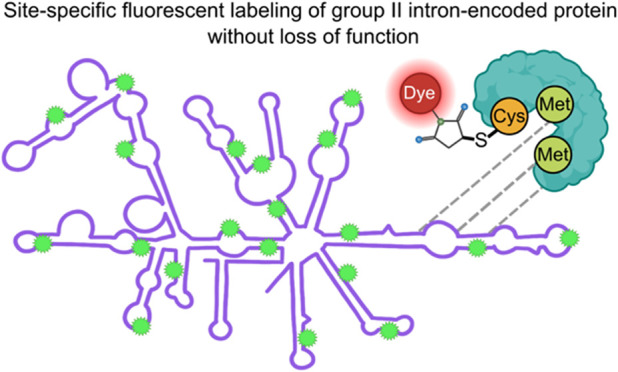

## Introduction

Group II introns are large catalytic RNAs capable of both self-splicing and site-specific integration into DNA, with deep evolutionary connections to the spliceosome and retroelements. Structural variations define three major classes of group II introns: IIA, IIB, and IIC.^1–3^ They share a conserved secondary structure consisting of a central core from which six RNA domains radiate outward.^4–6^ Domain V functions as the catalytic core of the ribozyme, coordinating magnesium ions (Mg^2+^) that mediate the transesterification reactions required for splicing.^7–11^ Interactions among the domains give rise to a complex tertiary structure that positions a bulged adenosine in domain VI (DVI) within the ribozyme’s active site.^12,13^ In addition to their highly structured RNA, many bacterial group II introns contain an open reading frame within domain IV (DIV) that encodes a multidomain intron-encoded protein (IEP).^14–16^ The IEP typically includes a conserved reverse transcriptase (RT) domain, which contains the fingers and palm subdomains, and a less conserved X domain, often referred to as the maturase or thumb domain.^15,17–20^ The catalytic YADD motif within the RT domain serves as the active site for cDNA synthesis during retromobility. Aspartate residues in this motif coordinate Mg^2+^ ions, which are essential for catalysis.^16,21–23^ IEPs also contain a less well-defined DNA-binding domain (DBD). Group IIA and IIB introns typically encode a C-terminal endonuclease (En) domain that facilitates DNA cleavage to prime cDNA synthesis during retromobility.^24^ In contrast, group IIC introns lack this En domain.^25^

Group II introns catalyze splicing by recognizing exon sequences through base pairing between exon-binding site (EBS) elements in the intron RNA and intron-binding site (IBS) motifs in the flanking exons. The EBS1–IBS1 interaction (typically 6–8 nucleotides) defines the 5′ splice site, while the γ–γ′ interaction within the intron and the EBS3–IBS3 interaction with the 3′ exon specify the 3′ splice site.^3,26–32^ These conserved RNA–RNA interactions are essential for precise intron excision and exon ligation. Many group IIA and IIB introns can generate lariat products through ribozyme-mediated, RNA-only self-splicing *in vitro*. In these reactions, a bulged adenosine in domain VI initiates nucleophilic attack at the 5′ splice site, forming a lariat–3′ exon intermediate. In the second step, the 5′ exon attacks the 3′ splice site, resulting in ligation of the exons.^33–35^ In contrast, group IIC introns primarily undergo splicing via hydrolysis under similar conditions, bypassing lariat formation and yielding a linear intron RNA.^8,36,37^ Unlike other group II introns, IIC introns rely on their intron-encoded protein to restore lariat formation during self-splicing. The IEP interacts with domain VI, ensuring proper positioning of the bulged adenosine within the catalytic RNA core of domain V.^12,13^ This interaction facilitates efficient 2′–5′ phosphodiester bond formation, enabling the branching reaction and promoting completion of the splicing pathway.^38,39^

Group II intron retromobility begins with the formation of an intron lariat-IEP ribonucleoprotein (RNP) complex. This complex is generated during IEP-facilitated intron splicing, during which the IEP remains tightly associated with the excised lariat product.^18,40^ The intron–IEP RNP identifies its DNA target site through base pairing between EBS sequences in the intron RNA and complementary IBS motifs in the DNA.^15,25,41,42^ The intron ribozyme catalyzes reverse splicing of the RNA into one strand of the DNA target, followed by IEP-mediated cleavage of the opposite strand via its endonuclease domain. This cleavage generates a free 3′–OH on the DNA, which primes reverse transcription of the intron RNA into cDNA. Integration is completed by host DNA repair pathways.^23,24,43^ In this study, we examine a group IIC intron, which lacks the En domain and instead relies on lagging-strand DNA fragments generated during replication to prime cDNA synthesis and integration.^44–46^

While the biochemical steps of mobility are increasingly well-characterized, the conformational changes that coordinate these steps have remained less clear. Recent cryo-electron microscopy (cryo-EM) studies have revealed how the intron-encoded protein orchestrates RNA structural transitions during group II intron splicing and mobility. During forward splicing, domain VI positions the bulged adenosine branchpoint near the catalytic core through interaction with the IEP, promoting lariat bond formation. After the first step, DVI shifts away from the catalytic site to position the 3′ splice site for exon ligation and lariat release. In reverse splicing, DVI begins oriented away from the intron active site core. It first aligns the lariat 3′–OH for attack at the DNA target site. Then, in the second step, the IEP re-engages to position the lariat bond for cleavage, joining the start of the intron to the 5′ DNA exon and completing integration.^12,13,23^ Together, these structural studies underscore the central importance of IEP-orchestrated DVI dynamics in coordinating the sequential steps of splicing and retromobility across the group II intron life cycle.

Motivated by these recent structural insights into IEP-guided intron dynamics, we set out to develop tools that would enable real-time visualization of IEP-intron interactions. Real-time visualization can provide valuable insights into transient intermediate states, which are often difficult to capture through static structural techniques, thereby addressing a significant gap in our mechanistic knowledge of RNA-protein interactions. One promising approach involves site-specific fluorescent labeling of the IEP to facilitate the dynamic monitoring of interactions and conformational changes during splicing and retromobility. To achieve this, we employed maleimide bioconjugation, which utilizes the thiol-reactivity of maleimide to form covalent bonds with available sulfhydryl groups in a reducing environment.^47^ When conjugated with a fluorescent dye, maleimides can be used to label the IEP; however, the presence of native cysteine residues compromises site specificity. To address this, we systematically replaced all native cysteine residues in the group IIC intron IEP from *Ta.it.*I1 (originating from the thermophile *Thermoanaerobacter italicus*) with methionine, thereby creating a cysteine-free variant of a group II intron IEP.

Our results demonstrate that removal of cysteine residues from the *Ta.it.*I1 IEP does not significantly impair IEP-dependent splicing via the lariat pathway or the formation of the resulting lariat-IEP RNP complex. Cysteine-free IEP variants also support both steps of reverse splicing during DNA integration. Notably, mutation of the cysteine in the thumb domain enhanced reverse transcription activity in our model system, potentially by increasing structural flexibility or improving enzyme-DNA interactions. Fluorescently labeled IEP mutants retained splicing activity, highlighting the IEP’s structural tolerance to modification. These findings support the utility of cysteine-free IEP variants as tools for tracking IEP-intron interactions and furthering mechanistic studies of group II intron function.

## Materials and Methods

### Protein Purification

The *Ta.it.*I1 intron-encoded proteins were purified using a pET11a expression vector transformed into Rosetta BL21 DE3 *Escherichia coli* cells. Cells were expressed in 1.5 L of Luria broth autoinduction media containing 100 μg/mL carbenicillin and 20 μg/mL chloramphenicol.^48^ Cells were harvested at 5000*g* and resuspended in 22 mL of lysis buffer (20 mM Tris-HCl [pH 7.5], 300 mM NaCl, 10 mM imidazole, and 5 mM β-mercaptoethanol) and treated with a 1/1000 dilution of 0.1 M PMSF protease inhibitor just before sonication. Cells were lysed on ice by sonication of 15 bursts of 8 s after 1 min pauses. The cell lysate was cleared by two consecutive spins at 5500 rpm (7000*g*) at 4 °C, added to equilibrated Ni-NTA resin, and allowed to bind rotating end-overend at 4 °C for 1 h. The resin was washed with lysis buffer at 500*g* until clear to remove unbound protein. The clean resin was resuspended in 15 mL lysis buffer, transferred to a 20 mL flow column, and washed with 30 mL wash buffer (20 mM Tris-HCl [pH 7.5], 300 mM NaCl, 20 mM imidazole, and 5 mM β-mercaptoethanol), 20 mL high salt buffer (2 M NaCl, 20 mM Tris-HCl [pH 7.5], 5 mM β-mercaptoethanol), and 30 mL lysis buffer. The protein of interest was eluted with 5 mL elution buffer (20 mM Tris-HCl [pH 7.5], 300 mM NaCl, 0.3 M imidazole, and 5 mM β-mercaptoethanol). The elution was desalted using a HiPrep 26/10 desalting column and ÄKTA start FPLC into desalt buffer (0.3 M NaCl, 20 mM Tris-HCl [pH 7.5], 2 mM β-mercaptoethanol). The desalted protein was concentrated using a 50 kDa Amicon cutoff spin filter (Millipore Sigma), and the concentration was determined using a Bradford assay on a Thermo Scientific Nanodrop One. The final protein is stored in 50% glycerol at −20 °C. If protein aggregates form, addition of 1 μL 4 M NaCl and gentle mixing may resolubilize protein precipitants.

### Protein Purification for Fluorescently Labeled Protein

Intron-encoded proteins used for maleimide–thiol fluorescent labeling were purified following the above procedure but without reducing agent in the buffers (β-mercaptoethanol).

### *In Vitro* RNA Transcription

Standard RNA transcription was performed with DNA template and T7 RNA polymerase.^49^ For *Ta.it.*I1 RNA, the 10× transcription buffer contains 50 mM MgCl_2_. After transcription, DNA is removed by DNase 1 digestion at 37 °C for 1 h. Following DNase digestion, transcripts were subjected to phenol extraction and ethanol precipitation to remove proteins in the sample. The resulting RNA pellets are resuspended in 0.5 mM EDTA and 1 mM sodium cacodylate [pH 6.5] and stored at −20 °C.

### *In Vitro* Splice Assay

Standard splicing assays are performed with 1 μg of *Ta.it.*I1 RNA and either a titration or a 55× excess of IEP in a 2× splice buffer (2× concentration is 10 mM MgCl_2_, 100 mM Tris-HCl [pH 7.5], 1 M NH_4_Cl, 20 mM DTT). RNA input control is prepared in water and not heated to inhibit self-splicing. Each splice reaction is incubated at 45 °C for 15 min and the reaction is stopped with the addition of a stop solution (final 1× concentration is 0.3 M NaOAc [pH 5.2], 5 mM EDTA, brought to volume with water) and immediately vortexed. The resulting reactions are phenol extracted with phenol-chloroform and ethanol precipitated with linear polyacrylamide as the carrier. The resulting dry pellet was resuspended in 2× formamide dye (2× concentration is 2 mM EDTA in formamide) and water and boiled at 90 °C for 3 min to denature the RNA. The samples are spun down at 20,000*g* for 1 min and resolved on a 4% urea denaturing PAGE (19:1 acrylamide:bis-acrylamide, 7 M urea) in tris-borate EDTA running buffer. The gel was stained in ethidium bromide and imaged in a Syngene G:Box. Splice assay gel images were analyzed and quantitated using ImageJ (Fiji). Raw gel files were loaded into the software, and a rectangular selection was drawn around each lane to isolate the region of interest. The intensity profile of each lane was then plotted, and the area under each peak corresponding to individual bands was measured. For each lane, the areas of all detected bands were summed to obtain a total signal value. The area of each individual band was then divided by this total to calculate the relative proportion of each product. These normalized ratios are reported in the figure plots.

### Reverse Transcription Assay

The Invitrogen Enzchek Reverse Transcriptase Assay Kit was used to determine the reverse transcription activity of each protein. Each protein was diluted 1:100 in dilution buffer (50 mM Tris-HCl [pH 7.5], 2 mM DTT, 20% glycerol). 10 μL of each diluted protein was transferred to 40 μL of annealed poly­(A) ribonucleotide template and oligo d­(T)_16_ primer diluted 200-fold in polymerization buffer (60 mM Tris-HCl, 60 mM KCl, 8 mM MgCl2, 13 mM DTT, 100 μM dTTP [pH 8.1]). The reaction was incubated at 25 °C for 25 min. Each reaction was quenched with 4 μL of 200 mM EDTA. 25 μL of each reaction was transferred to a 96 well plate, and 173 μL of Picogreen reagent diluted 345-fold in 1× TE (10 mM Tris-HCl [pH 7.5], 1 mM EDTA) was added. The samples were incubated at 25 °C for 3 min. The relative fluorescence was measured with a BioTek Synergy H4 Hybrid plate reader. Dilution buffer without protein was used as a negative control.

### Fluorescent Oligonucleotide Substrate

Retromobility assays were performed with a fluorescent DNA oligonucleotide with a 5′ end DNA stem-loop labeled with Cy5 and a 3′ end hairpin labeled with FAM that was synthesized by IDT. The sequence of the substrate was

Cy5GCAGTCTAAAAGTAATTTTAGACTGCTTTTTTATTTTTTCCGCGCTTCGGCGCGG. The underlined thymine indicates the position of the FAM label.

### Retromobility Assay

RNPs with each protein were formed by the lariat splicing reaction as described in the splice assay method at 45 °C for 10 min. Assembled RNPs were incubated at 37 °C for 15 min with 600 nM fluorescent DNA substrate with and without 10 μM dATPs. 600 nM fluorescent DNA substrate in water was also incubated as a control. All reactions and controls were phenol-extracted and ethanol precipitated. Extracted nucleic acid was resuspended in 2× EDTA formamide loading buffer (2 mM EDTA in formamide) and water. Samples were heated at 90 °C for 2 min before being resolved on 4% urea denaturing PAGE (19:1 acrylamide/bis-acrylamide, 7 M urea) in tris-borate EDTA running buffer. Fluorescence was imaged using a GE Amersham Typhoon imaging system.

### Retromobility Time Course Assay

RNPs with each protein were formed by the lariat splicing reaction as described in the splice assay method at 45 °C for 10 min. Assembled RNPs were incubated at 37 °C for 2, 5, and 10 min with 600 nM fluorescent DNA substrate and 10 μM dNTPs. 600 nM fluorescent DNA substrate in water was also incubated as a control. The reaction was stopped with the stop solution used in the splice assay method. All reactions and controls were phenol-extracted and ethanol precipitated. Extracted nucleic acid was resuspended in 2× EDTA formamide loading buffer (2 mM EDTA in formamide) and water. Samples were heated at 90 °C for 4 min before being resolved on 4% urea denaturing PAGE (19:1 acrylamide/bis-acrylamide, 7 M urea) in tris-borate EDTA running buffer. Fluorescence was imaged using a GE Amersham Typhoon imaging system.

### Fluorescent RNA Labeling via Transcription

Standard transcription was performed with DNA template and T7 RNA polymerase.^49^ 10 mM rNTPs were used with addition of 20 μM FAM-labeled UTPs. To optimize for *Ta.it.*I1 RNA, the 10× transcription buffer for fluorescently labeled RNA contains 20 mM MgCl_2_. Transcription occurs at 37 °C for 2 h. After transcription, DNA is removed by DNase 1 digestion at 37 °C for 1 h. Following DNase digestion, transcripts were subjected to phenol extraction and ethanol precipitation to remove proteins in the sample. The resulting RNA pellets are resuspended in 0.5 mM EDTA and 1 mM sodium cacodylate [pH 6.5] and stored at −20 °C.

### Maleimide–Thiol Fluorescent Protein Labeling

Freshly eluted recombinant protein purified as described in the protein purification for fluorescently labeled protein was buffer-exchanged into 0.3 M NaCl and 20 mM Tris-HCl [pH 7.5] using a HiPrep 26/10 desalting column (Cytiva) on an ÄKTA Go FPLC system (GE Healthcare). Protein concentration was determined using a Bradford assay measured on a Thermo Scientific Nanodrop One spectrophotometer. To reduce cysteine residues for fluorophore labeling, a 10-fold molar excess of tris­(2-carboxyethyl)­phosphine (TCEP) to the protein concentration determined by Bradford assay was added to each protein sample. Typical protein concentrations for labeling were about 8 μM and the TCEP concentration to reduce available cysteine residues was about 80 μM. The mixture was vortexed and incubated at room temperature for 5 min and TCEP was not removed from the sample before labeling. Alexa Fluor 647 C2-maleimide (AF647; Invitrogen; Excitation: 651/Emission: 671, Extinction coefficient: 265,000 cm^–1^ M^–1^) was prepared as a 10 mM stock solution in DMSO and stored at −20 °C until use. For labeling, the dye was diluted to 1 mM in 0.3 M NaCl and 20 mM Tris-HCl [pH 7.5] and added in excess to the reduced protein. Labeling reactions were incubated either at room temperature for 2 h with gentle rotation or overnight at 4 °C. Excess AF647 and TCEP was removed using a HiPrep 26/10 desalting column (Cytiva) on the ÄKTA Go system and buffer exchanged into 0.3 M NaCl and 20 mM Tris-HCl [pH 7.5]. The labeled protein was concentrated using a 50 kDa Amicon cutoff centrifugal filter (Millipore Sigma). Final protein concentration was determined by absorbance at 280 nm (*A*_280_) using the Nanodrop One since Bradford assay was not suitable to measure the colored protein sample. The labeling efficiency of each protein was calculated by measuring *A*_max_ using a Thermo Scientific Nanodrop One spectrophotometer divided by the extinction coefficient of the conjugated dye multiplied by the protein concentration. The labeling efficiency is reported in moles of dye/protein. Labeled protein was aliquoted in 50 μL volumes into PCR tube strips, flash-frozen in liquid nitrogen, and stored at −80 °C until use.

### Protein–RNA Native Shift Assay

Native PAGE mobility shift assays were performed using FAM-labeled RNA (1.8 μM final concentration) incubated with a titration of Alexa Fluor 647 labeled IEP protein in 5× splicing buffer. The 5× concentration of the splicing buffer consisted of 25 mM MgCl_2_, 250 mM Tris-HCl [pH 7.5], 2.5 M NH_4_Cl, and 50 mM DTT. Protein was added at 5× and 15× molar excess relative to RNA, with a constant RNA input of 1.8 μM and protein input up to 30 μM. Each RNP assembly reaction and corresponding protein control was incubated at 45 °C for 15 min, followed by brief centrifugation. To 10 μL of each reaction, 3 μL of 80% glycerol was added as loading buffer. Samples were loaded onto a prechilled 5% native polyacrylamide gel and electrophoresed in 0.5× Tris-borate buffer supplemented with 5 mM MgCl_2_ (included in both gel and running buffer to preserve RNA structure). Electrophoresis was performed at 12 W for 1 h, followed by 15 W for 2 h at 4 °C in a cold room, cycling the running buffer every hour. Fluorescent signals were visualized using a Typhoon imaging system (GE Amersham). Additionally, the gel was stained overnight in colloidal Coomassie G-250 stain. The gel was destained in water followed by visualization by a Typhoon imaging system (GE Amersham).

## Results

### Cysteine-Free IEP Variants Retain Maturase Activity in Splicing Reaction

Group II intron-encoded proteins function as reverse transcriptases and contain three structural domains: fingers, palm, and thumb. The palm region includes the catalytic YADD motif that is essential for reverse transcription.^15–18^ The thumb contains the X domain and a less defined DNA-binding domain (Figure 1A). To enable site-specific fluorescent labeling, we used maleimide dyes that react with cysteine thiol groups.^47^ This strategy required removal of all native cysteines from the IEP. In the *Ta.it.*I1 IEP, three native cysteines were identified: two located near the YADD motif in the RT domain and one located in the thumb domain near the C-terminus (Figure 1B). Sequence alignments showed that the cysteines following the YADD motif and in the thumb domain are conserved in group IIC IEPs. This conservation suggests they may contribute to enzymatic function (Figure 1B).

**Figure 1:**
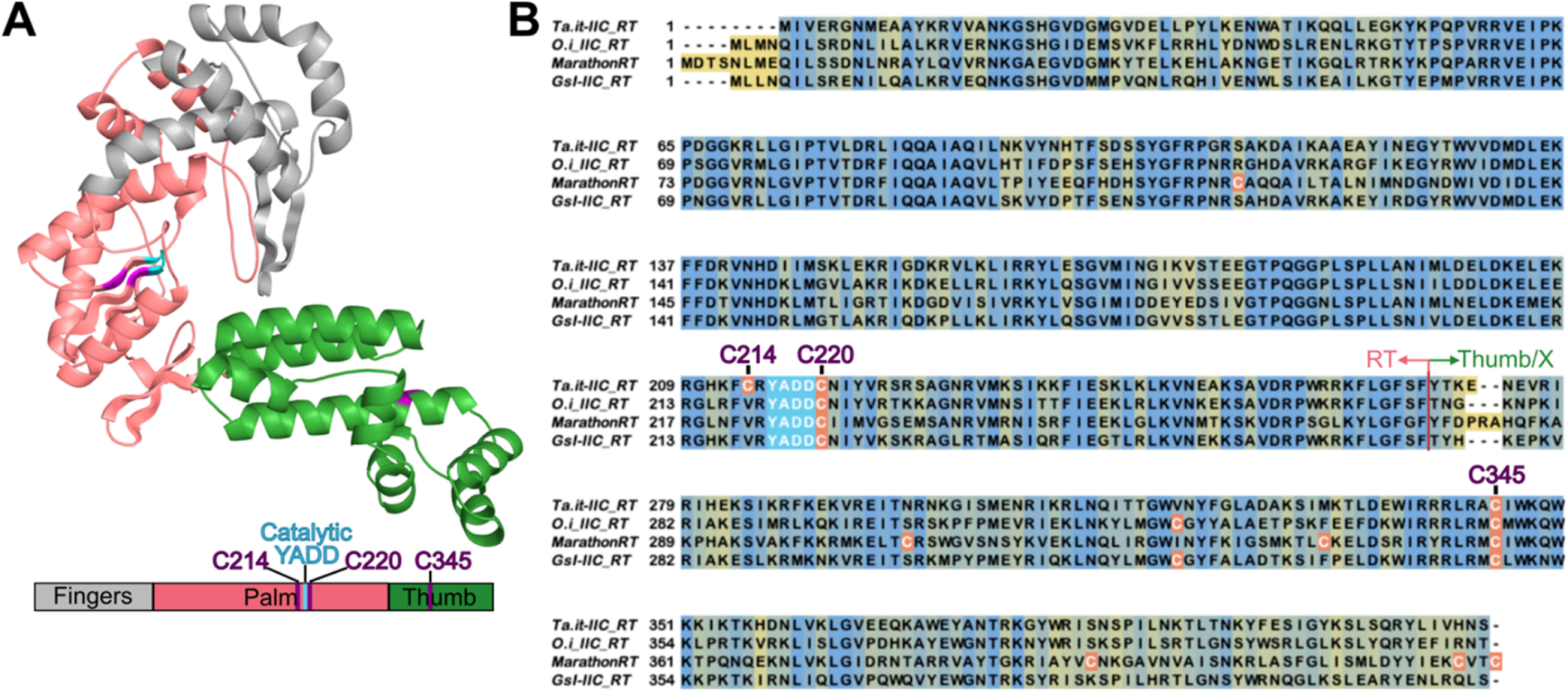
Group IIC intron-encoded protein sequence alignment showing conserved domains. (A) α-fold3 representation of *Ta.it.*I1 IEP colored by canonical RT regions of fingers (gray), palm (light pink), and the X/DBD regions of the thumb (green). The catalytic YADD resides within the palm region of the RT domain, colored in cyan, and the cysteines are colored in purple. The box below the structure represents the sequence of the RT and the location of the cysteine residues (purple). (B) Sequences of four group IIC intron-encoded protein sequences (*Ta.it.* RT, *O.i.* RT, MarathonRT, *GsI* RT) showing conservation between the RT domain (light pink arrow) and X and DBD domains (green arrow). More yellow indicates less conservation while more blue indicates more conservation. The catalytic YADD is highlighted in cyan and all endogenous cysteine residues for each RT are highlighted in orange with their names in purple.

Native cysteines in the *Ta.it.*I1 IEP were systematically replaced with methionine using an N-terminal His_8_-SUMO-IEP expression construct to generate a panel of cysteine-substituted variants (Figure 2A). Each mutant was expressed in *E. coli* using autoinduction and purified by affinity chromatography with nickel-agarose resin (Figure 2B). A catalytically inactive control IEP, in which the YADD motif in the RT domain was mutated to YAAA, was also expressed and purified. Substitution of the aspartic acid residues in the YADD motif with alanine prevents magnesium ion binding and blocks reverse transcriptase activity.^16,22,23,50^ The splicing activity of wild-type (WT), cysteine-mutated, and YAAA IEP variants was evaluated using an *in vitro* IEP-dependent splicing assay with *Ta.it.*I1 group II intron RNA. In the presence of the IEP, the intron follows the lariat splicing pathway to generate the RNP complex (Figure 2C). This assay measured the ability of each IEP variant to bind the intron RNA and promote lariat splicing. Activity was compared to WT IEP as a control (Figure 2D). All cysteine-mutated IEPs retained splicing activity, with 50% to 60% of the RNA following the lariat pathway. This is comparable to the average level of lariat formation observed with WT IEP (Figure 2D, right). These results show that substituting native cysteines with methionine does not impair maturase activity and supports the use of maleimide-based labeling strategies. An additional cysteine-to-serine *Ta.it.*I1 IEP mutant was purified to assess the impact of splicing with another suitable residue with more hydrophilic group (Figure S3). While purifications of the complete cysteine-to-methionine and cysteine-to-serine were nearly identical, the splicing activity with the cysteine-to-serine IEP mutant produced less lariat in comparison to the complete cysteine-to-methionine mutant (Figure S3).

**Figure 2:**
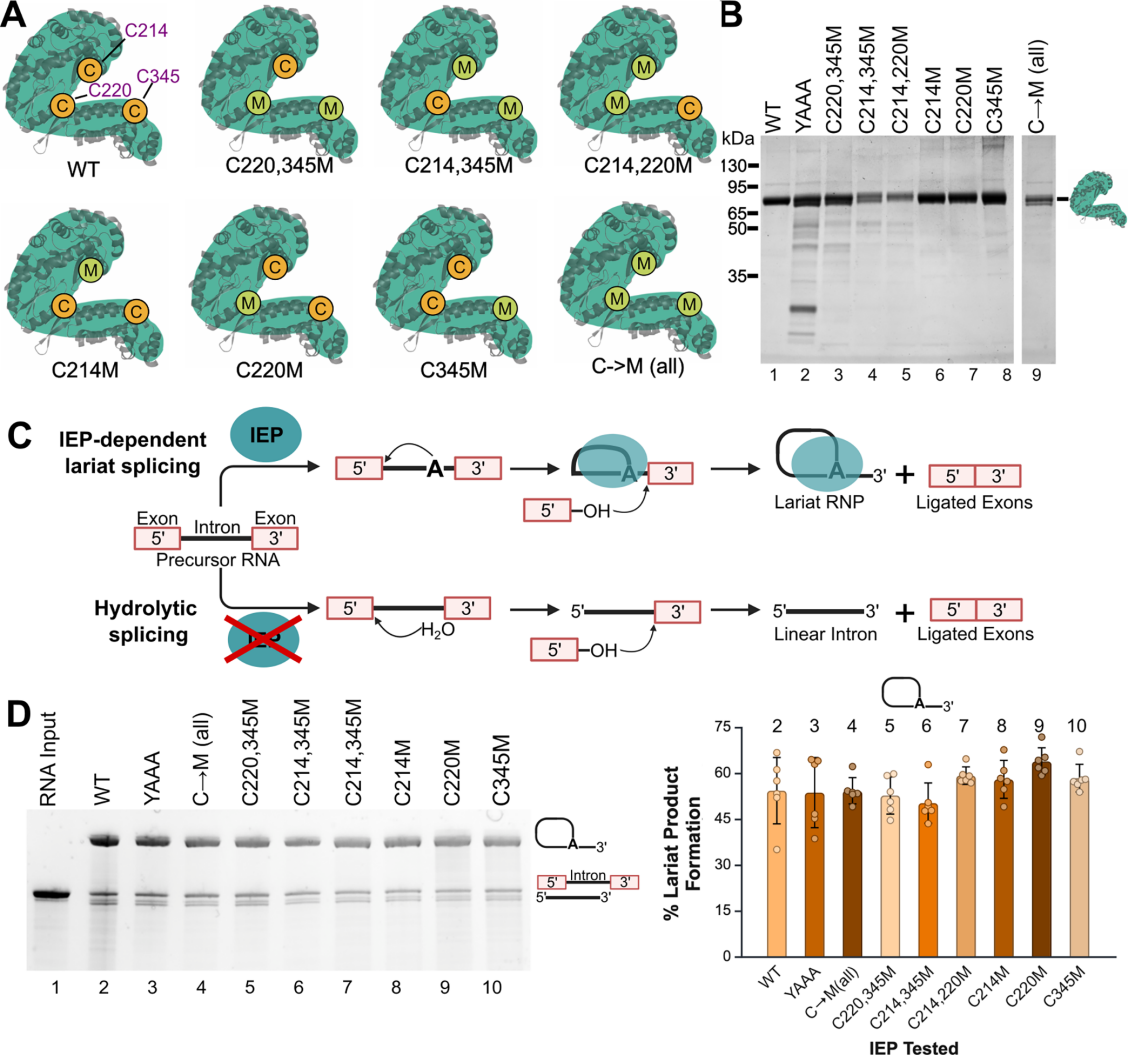
Purification and *in vitro* splicing activity of cysteine-mutated IEPs. (A) Cartoon depiction of the mutant IEPs and the sites of cysteine mutagenesis with their respective names below. (B) SDS-PAGE of purified IEP cysteine mutants, WT, and YAAA. (C) Schematic of the two competing splicing pathways of *Ta.it.*I1 intron with and without IEP. (D) 4% PAGE analysis of *in vitro* splicing activity of *Ta.it*.I1 intron RNA with IEP cysteine mutants, YAAA mutant, and WT under standard splicing conditions. The bar graph (right) compares the average percent lariat formed with each IEP cysteine mutant and YAAA with WT IEP. The mean was calculated with five replicates, and the error bars represent the standard deviation (SD). The numbers above the bar graph correspond with their respective gel lane. Uncropped versions of (B, D) is in Figures S1 and S2, respectively.

### Differential Effects of Cysteine Mutations on IEP RT Activity

After confirming that IEP variants with cysteine mutations, including a cysteine-free construct, supported group IIC RNP formation, we next evaluated their reverse transcriptase (RT) activity. RT activity is essential for group II intron mobility, as it converts the reverse-spliced intron RNA into cDNA for genomic integration.^15,16,24,51^ This activity is catalyzed by the YADD motif in the RT domain of the IEP.^16,21,22,52^ We used a poly­(rA)/oligo­(dT)_16_ primer extension assay with dTTP to measure RT activity. Picogreen fluorescence was used to detect RNA–cDNA duplex formation (Figure 3A). WT IEP produced a strong signal and served as the positive control. The catalytically inactive YAAA mutant was used as a negative control. This mutant generated minimal signal, due to residual Picogreen incorporation into unreacted poly­(rA)/oligo­(dT)_16_ duplex. This baseline signal matched the signal observed in no-IEP controls, confirming the absence of catalytic activity in the negative control (Figure 3B).

**Figure 3:**
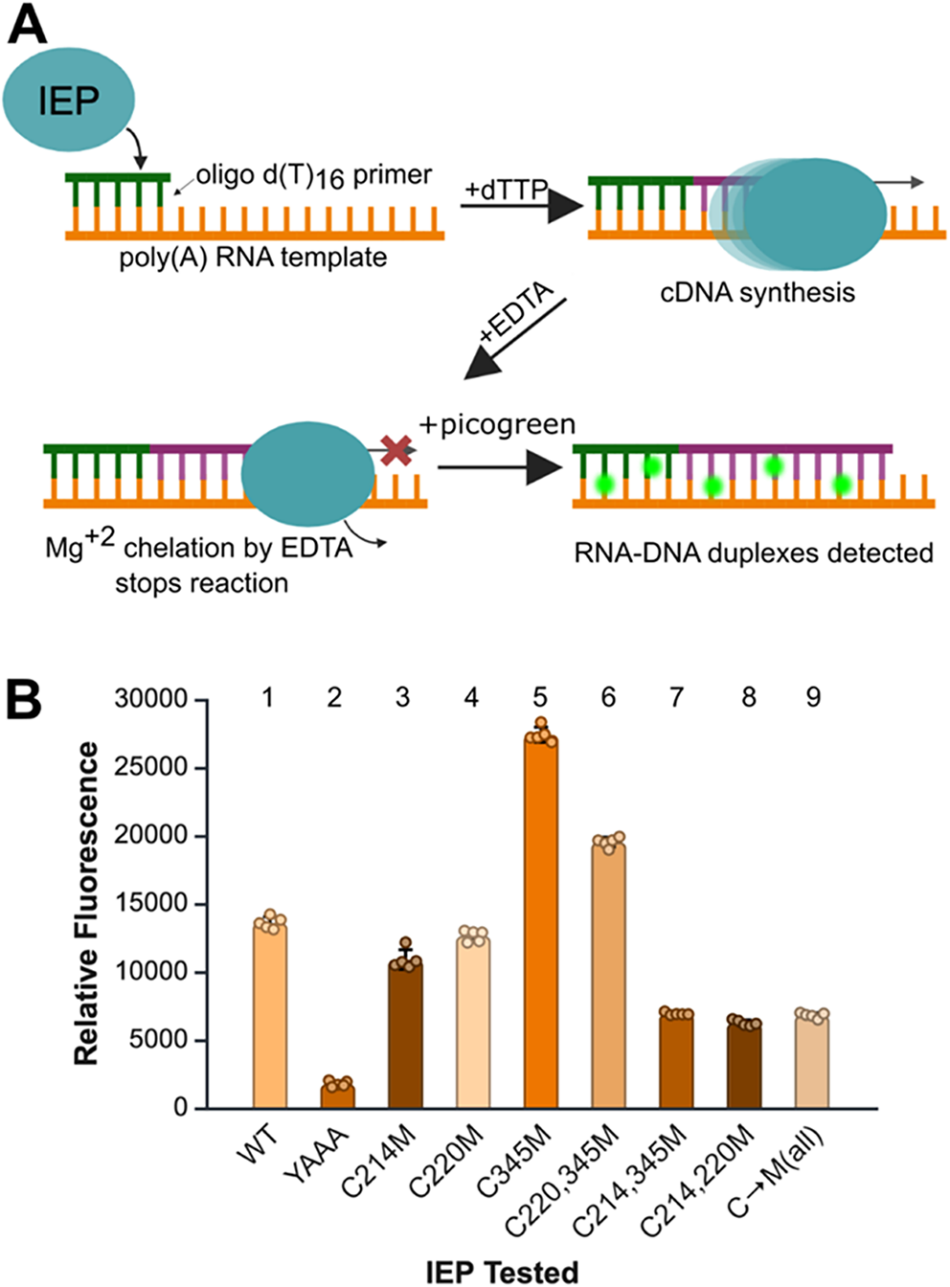
*In vitro* reverse transcription activity of cysteine-mutated IEPs. (A) Schematic of the reverse transcription assay. (B) Bar graph of average relative fluorescence measured in each cysteine-mutated IEP, WT, and YAAA. The average fluorescence was calculated with five replicates, and the error bars represent the SD.

We first examined the effects of single C-to-M point mutations in the IEP. The C214M mutation resulted in a ∼25% reduction in RT activity. Mutation of the more conserved C220 position, located downstream of the YADD motif (Figure 1B), retained ∼90% of WT activity. In contrast, the C345M mutation in the thumb domain exhibited a striking 2-fold increase in RT activity (Figure 3B, compare lanes 1 and 5). Next, we investigated whether the strong stimulatory effect of C345M could compensate for the decreased activity caused by mutations near the RT active site. The C214,345M double mutant failed to rescue the reduced activity of C214M point mutant, and further decreased activity to 43% of WT levels. Conversely, the C220,345M double mutant showed an increase to 150% of WT activity, though this was still lower than the C345M single mutant (Figure 3B, compare lanes 5 and 6). Completing the analysis, we examined the combined effects of mutating both YADD-proximal cysteines. The C214,220M double mutant retained 38% of WT activity, lower than either single mutation. Finally, we generated a cysteine-free IEP by mutating all three native cysteines to methionine (C214,220,345M). RT activity of the triple mutant was comparable to that of any C214M-containing mutant (Figure 3B, compare lanes 3, 7–9). These results suggest that C214 negatively regulates RT activity in the *Ta.it.*I1 IEP, and that this inhibition cannot be compensated by the stimulatory effect of the C345M mutation.

### Cysteine-Mutated IEPs Support Reverse Splicing and Intron Integration

The retromobility activity of WT, cysteine-mutated, and YAAA mutant IEPs was evaluated using an *in vitro* fluorescent assay. RNPs were assembled by incubating each IEP variant with WT *Ta.it.*I1 intron RNA under splicing conditions, as described previously. This allowed us to test whether differences in IEP function, particularly RT activity, impact DNA integration. We previously developed a fluorescence-based assay to monitor *Ta.it.*I1 intron integration into a dual-labeled DNA substrate.^52^ The substrate includes a Cy5 label at the 5′ end and a fluorescein-dT (FAM) label at the 3′ end, allowing discrimination of reverse splicing products by fluorescence. In the absence of dNTPs or RT activity, the reaction stalls after the first step, producing a 3′ lariat–DNA intermediate.^52–54^ Addition of dATP enables limited cDNA synthesis and promotes completion of the second reverse splicing step, resulting in full integration.^52^

RT assays showed that cysteine mutation at C214, near the YADD motif, reduces RT activity. To test whether this reduction affects mobility, RNPs were incubated with the labeled DNA substrate in the presence or absence of dATP (Figure 4A). The DNA target includes four dT residues at the start of the template, limiting cDNA synthesis to four nucleotides and providing a controlled readout of RT activity. All IEP variants, including those with reduced RT activity, produced the 3′ lariat-DNA intermediate at levels similar to WT in the absence of dATP (Figure 4C, left). When dATP was added, all cysteine-mutated IEPs completed the second step of reverse splicing and initiated cDNA synthesis at levels comparable to WT (Figure 4C, right). These results show that minimal RT activity is sufficient to drive intron integration *in vitro*. Because the RT assays measured activity based on a small poly­(A) template lacking complex secondary structure, the cDNA synthesis activity of the C345M and C214,220M during retromobility was evaluated with addition of dNTPs to promote full-length cDNA synthesis (Figure 4D). Although the minimal RT activity in C214,220M was sufficient to complete integration, full-length intron cDNA synthesis was stalled with about 15% of the RNA being integrated with complete cDNA synthesis. The C345M mutant exhibited a slightly increased rate of full-length cDNA production compared to WT IEP but was not the 2-fold increase observed in the RT assay (Figure 4D, right).

**Figure 4:**
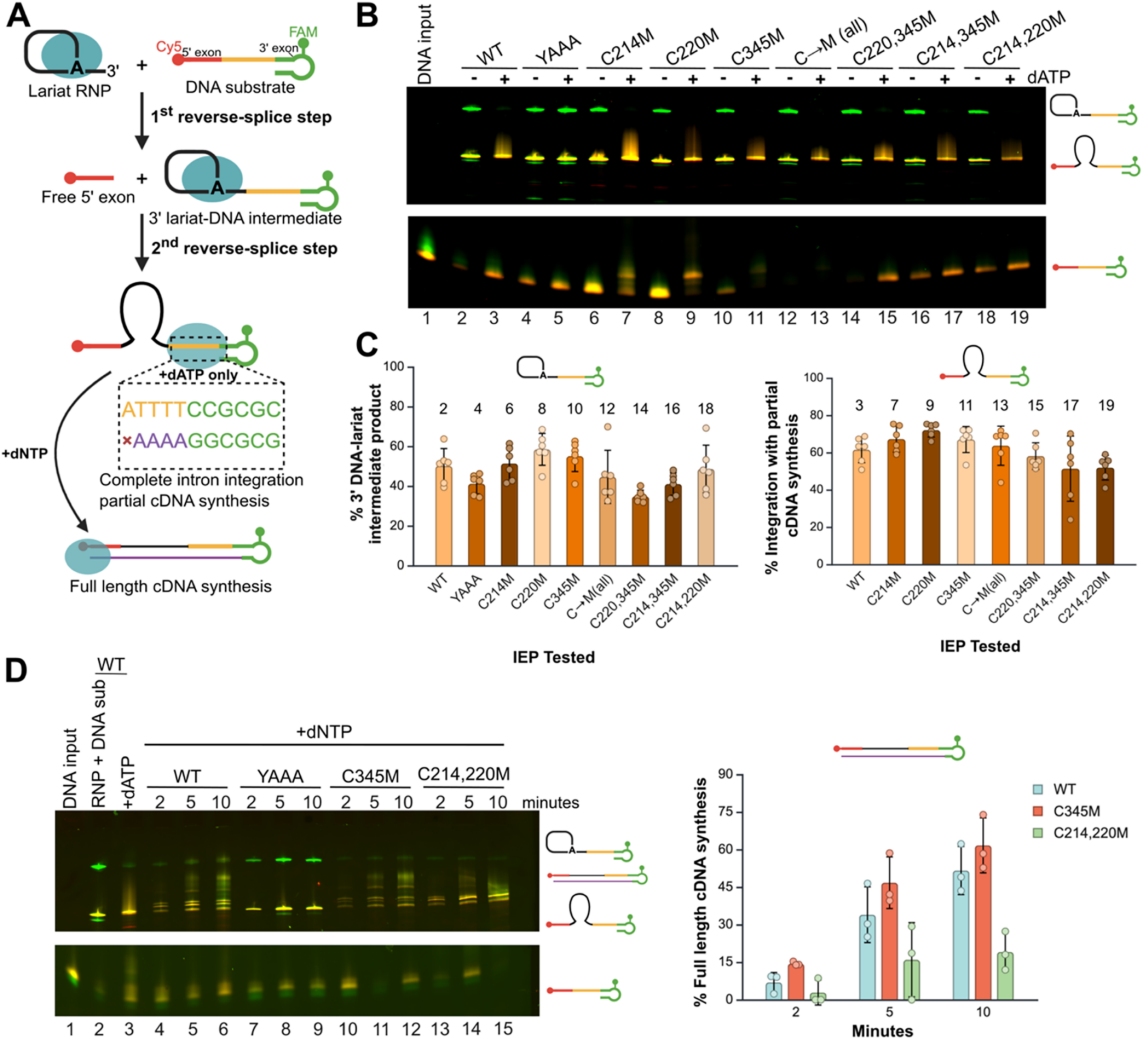
*In vitro* retromobility of cysteine-mutated IEPs. (A) Schematic of the retromobility assay. The dashed lines represent a close-up visualization of limited cDNA synthesis with addition of dATPs. (B) Cysteine mutants, YAAA, and WT RNP were tested for integration into the fluorescently labeled DNA substrate with and without dATP. Green bands indicate FAM detection of the 3′ exon only. Yellow bands indicate detection of both Cy5 and FAM, showing that the 5′ and 3′ exons of the DNA substrate are detected together. (C) A bar graph (left) compares the average percent of 3′ lariat-DNA intermediate produced (green band only) of IEP cysteine mutants and YAAA with WT. Another bar graph (right) compares the average complete integration product with limited cDNA synthesis by dATP addition of IEP cysteine mutants and YAAA with WT (yellow band). The averages were calculated with five replicates, and the error bars represent the SD. The numbers above the columns of each bar graph correspond to the band lane in the above retromobility gel in (B). (D) A time course analysis of full-length cDNA synthesis was performed comparing C345M and C214,220M with WT after addition of dNTP. The band colors follow the same as in (B), however the smearing of the yellow indicates different lengths of synthesized cDNA. The bar graph (right) shows the average full-length product at each time point for C345M and C214,220M relative to WT. Averages were calculated from three replicates, and error bars represent the standard deviation (SD). Uncropped versions of panels (B, D) are shown in Figures S4 and S5, respectively.

### Fluorescent Labeling of Cysteine-Mutated IEPs Does Not Disrupt Splicing Activity

After validating the activity of each cysteine-mutated IEP, we tested whether selected mutants could be used for fluorescent labeling to monitor RNA binding. Based on previous structural studies, DIV of the intron RNA is predicted to mediate interactions with the IEP.^13,43,55,56^ To enable labeling near this region, we used the C220,345M double mutant as a general labeling control and generated an additional variant, E271C, in which a native glutamate near the proposed DIV interaction site was replaced with cysteine (Figure 5A).^13^ The E271C IEP variant contains methionine substitutions at all three native cysteine positions. Each protein was labeled with Alexa Fluor 647 (AF647) maleimide dye, which covalently reacts with thiol groups on the engineered cysteine.^47,57^ Labeling was efficient, and fluorophore addition did not affect protein solubility (Figure 5B). The labeling efficiency for C220,345M was 60% and the labeling efficiency for E271C was 90%. The presence of lower molecular weight bands in the overall protein sample indicates degradation products that do not adversely affect protein activity. Wild-type IEP, which retains all native cysteines, was not labeled but served as a positive control for lariat splicing. To assess whether labeling affected IEP function, splicing activity was measured using an *in vitro* assay with *Ta.it.*I1 intron RNA.^52^ Both labeled and unlabeled IEP mutants supported lariat splicing with no significant difference in activity compared to WT IEP (Figure 5C). These results show that site-specific fluorescent labeling of engineered cysteine residues does not impair splicing function and supports the use of labeled IEPs for downstream RNA-binding studies.

**Figure 5:**
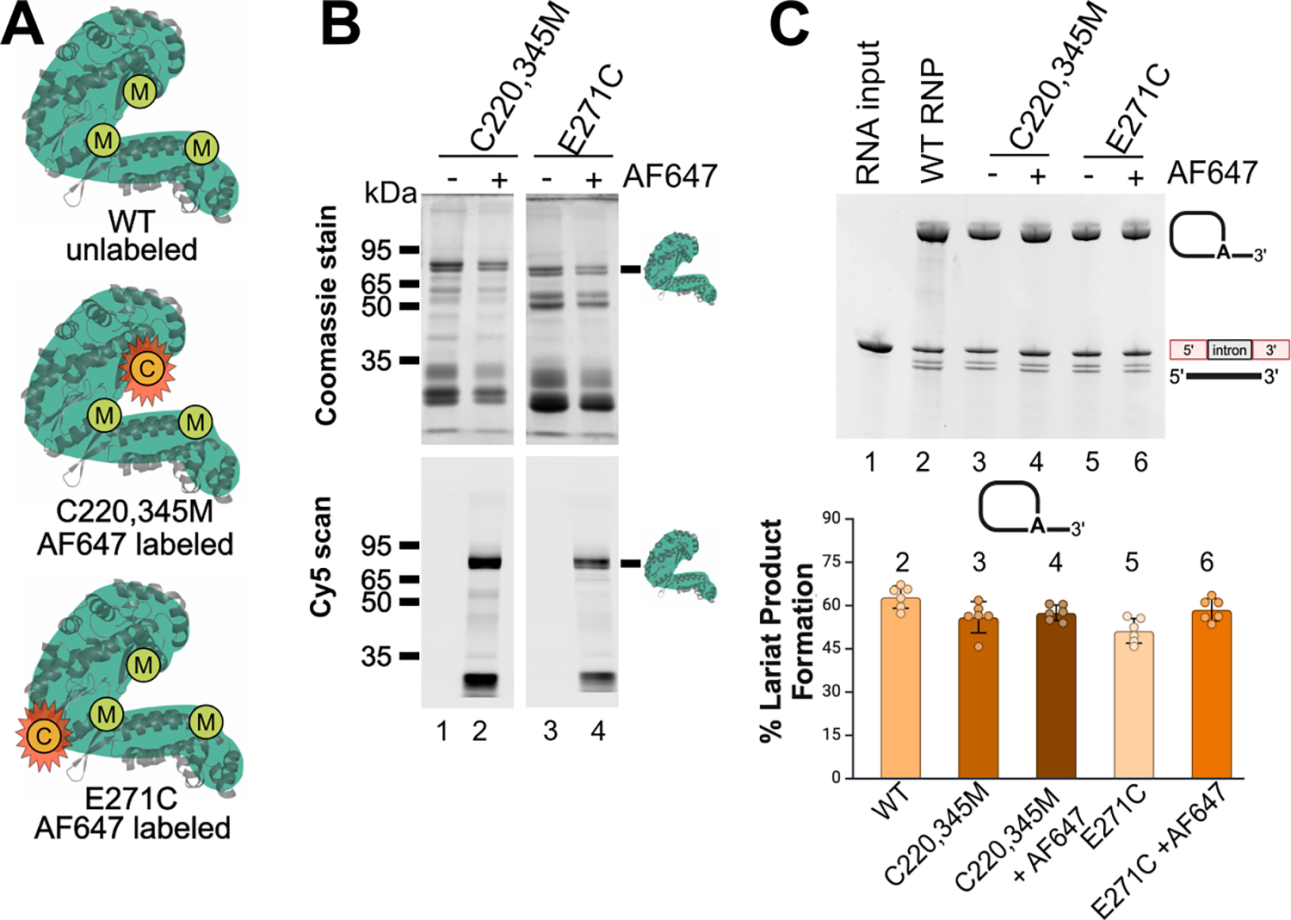
Functional maleimide labeling of cysteine-mutated IEPs (A) Cartoon depiction of representative cysteine-mutated IEP’s labeled with AF647 compared to WT IEP. (B) SDS-PAGE comparing cysteine mutant IEPs samples before and after fluorescently labeling with a functional maleimide containing AF647. Coomassie stains (top gel) show the protein population in each sample, with the main purified component at 68.6 kDa. Cy5 scan (bottom gel) is the same gel but scanned for detection of IEP labeled with AF647, showing the same purified labeled band at 68.6 kDa. (C) 4% Denaturing PAGE analysis of *in vitro* splicing activity of *Ta.it.*I1 intron RNA with labeled IEP cysteine mutants compared to unlabeled and WT under standard splicing conditions. The bar graph (right) compares the average lariat produced between labeled and unlabeled IEP, which is also compared back to WT. The average lariat value was calculated from five replicates, and error bars represent the standard deviation (SD). The numbers above the bar graph correspond to the respective gel lanes. Uncropped versions of panels (B, C) are shown in Figures S6 and S7, respectively.

### AF647-Labeled IEP Forms RNPs with FAM-Labeled RNA

After confirming that fluorescent labeling did not impair splicing activity, we tested whether labeled IEPs could associate with FAM-labeled intron RNA to monitor RNP assembly under native conditions. Full-length intron RNA was generated by *in vitro* transcription in the presence of FAM-UTP, resulting in transcript-wide fluorescent labeling (Figure 6A). The incorporation of FAM did not affect splicing, as shown by denaturing PAGE analysis following titrations with AF647-labeled C220,345M and E271C IEP variants (Figure 6B).

**Figure 6:**
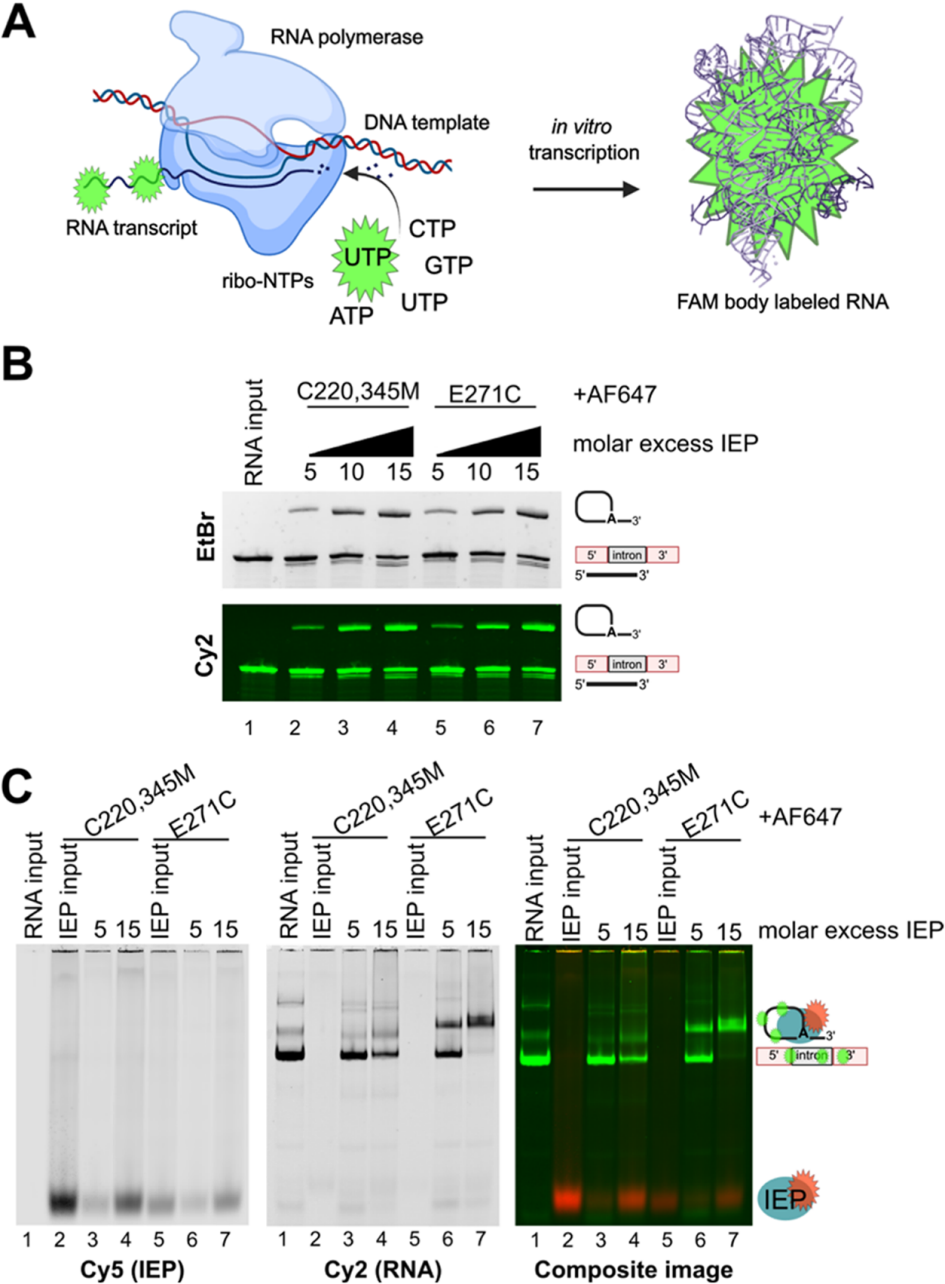
Functional AF647 IEP and FAM body-labeled intron RNA. (A) Cartoon representation of the fluorescent-labeled RNA method. FAM-labeled UTPs are supplemented to the *in vitro* transcription reaction to yield FAM body-labeled intron RNA. (B) 4% Denaturing PAGE analysis of *in vitro* splicing activity with FAM-labeled RNA with a titration of either AF647-labeled C220,345M or E271C IEP. The top gel is stained with ethidium bromide to show the RNA as it is spliced, and the bottom gel is a Cy2 scan to excite the FAM fluorophore, showing the retention of fluorescence after lariat splicing. (C) 5% Native PAGE analysis of *in vitro* splicing activity with FAM-labeled RNA comparing the RNP formation dynamics under low and high molar excess of AF647 C220,345M and E271C IEP. Left gel shows the Cy5 scan of the AF647 IEP present in the gel, middle gel shows the Cy2 scan of the FAM RNA present in the gel, and the right gel shows the composite image of the Cy2 and Cy5 scans showing how the bands overlap and interact. Uncropped versions of (B, C) is in Figures S8 and S9, respectively.

To assess RNP formation, FAM-labeled RNA was incubated with either low or high concentrations of AF647-labeled IEP, mirroring conditions used in the splicing assay. Native PAGE followed by fluorescence imaging revealed mobility shifts indicative of RNP assembly. At lower IEP concentrations (Figure 6C, red channel, lanes 3 and 6), the molar excess of the IEP was below the saturation point for RNP formation. Under these conditions, all available labeled IEP would be expected to bind RNA and form RNP complexes if fully active. Consistent with this expectation, no free labeled protein was detected in these lanes. This outcome matches the splicing assay at the same concentration where only low levels of lariat formation were observed (Figure 6B, lanes 2 and 5). At higher IEP concentrations (above the saturation point for IEP-dependent lariat–IEP complex formation), a distinct mobility shift in the FAM-RNA band was detected (Figure 6C, green channel, lane 7), consistent with stable RNP assembly. As expected for saturating conditions, excess free labeled protein was also observed migrating at the same position as the IEP input lanes. This condition corresponded with increased lariat formation in the splicing assay, confirming that saturated IEP levels promote efficient binding and catalysis. While the appearance of the complexes differs between native and denaturing PAGE due to their distinct electrophoretic environments, the matched concentrations between assays support consistent and specific RNP assembly. To confirm that the Cy5 signal represented intact labeled protein rather than degradation products, the native PAGE was stained with colloidal Coomassie (Figure S9). These results demonstrate that cysteine-labeled IEPs remain competent for RNA binding and RNP formation under native conditions. The labeled proteins retain full functionality as shown by fluorescent scans and Coomassie staining and enable direct visualization of RNP assembly, providing a powerful platform for future studies of group II intron binding dynamics and structural organization.

## Discussion

Group II introns are mobile ribozymes that play important roles in genome evolution and biotechnology.^41,43,58^ Their encoded proteins support multiple functions, including RNA folding, splicing, and reverse transcription.^20,39,59^ In this study, we show that cysteine-to-methionine mutants of the *Ta.it.*I1 group IIC IEP retain both maturase and reverse transcriptase activity and can be fluorescently labeled via maleimide–thiol conjugation without compromising function. Sequence alignments reveal that C220 and C345 are highly conserved among group IIC IEPs. C220 directly follows the catalytic YADD motif, suggesting a potential role in reverse transcription. C345 is the only cysteine present in the thumb domain of *Ta.it.*I1 IEP, whereas other group IIC IEPs, such as MarathonRT, contain multiple cysteines in this region.^60^ In contrast, group IIA and IIB intron IEPs contain well-defined C-terminal DNA-binding and En domains.^26^ The DNA-binding domain is characterized by a cluster of basic residues and an α-helix zinc finger-like motif, while the En domain contains a conserved His–Asn–His (H–N–H) motif interspersed with two cysteine pairs. The H–N–H motif coordinates a Mg^2+^ ion for catalysis, while the cysteine residues contribute to structural stability.^15,61^ Although group IIC IEPs lack a canonical En domain and well-defined DNA-binding motifs, conserved cysteine residues near the C-terminus appear important for proper protein folding and solubility. During purification, we encountered significant solubility issues and protein aggregation with several double cysteine-to-methionine mutants (C214,220M; C214,345M; and C220,345M). Additionally, the double cysteine-to-methionine mutants were more susceptible to degradation products in the sample. In contrast, single mutants (C214M, C220M, and C345M) remained soluble, and wild-type protein could be consistently purified without difficulty and minimal degradation. These observations suggest that these cysteines contribute to the structural stability of the *Ta.it.*I1 IEP, and that their loss may disrupt local folding or promote aggregation. Similar residues in other IIC IEPs may play analogous roles, particularly in proteins containing additional cysteines that could further influence solubility. To assess whether cysteine removal is broadly tolerated among group II intron-encoded proteins, we attempted to express the *T. elongatus* (*T.el*) IEP, which has been used in cryo-EM structural studies of group IIB RNPs.^12^ When we introduced a full set of Cys-to-Met substitutions, the protein was completely insoluble under standard purification conditions, precluding biochemical analysis (Figure S10). These results highlight variability in folding and solubility across group II IEPs and suggest that successful engineering of these proteins may require system-specific optimization.

We assessed maturase activity in all cysteine mutants. Maturase function refers to the ability of the intron-encoded protein to stabilize the intron RNA and promote proper RNA folding required for lariat formation during splicing.^20,58,59^ Despite mutations near the palm domain, which contains the catalytic YADD motif and contributes to RNA folding through Mg^2+^ coordination, all mutants retained full splicing activity.^16,22^ This indicates that the conserved cysteine residues are not essential for maturase function. While cysteine side chains can coordinate metal ions, they do not bind magnesium effectively. Magnesium is a hard Lewis acid that preferentially interacts with oxygen-rich ligands, such as the carboxylate groups of aspartic and glutamic acids, due to their electrostatic and geometric compatibility.^62^ In contrast, cysteine’s soft sulfur donors are poorly suited for Mg^2+^ binding and are rarely observed in magnesium coordination spheres.^62,63^ Structural surveys of metalloproteins confirm this distinction, with Mg^2+^ typically coordinated by aspartates or water molecules rather than cysteines.^64,65^ These data further support the conclusion that the conserved cysteines in *Ta.it.*I1 IEP are not directly involved in metal coordination for maturase activity.

While maturase function remained intact, cysteine mutations had distinct effects on the reverse transcription activity of the *Ta.it.*I1 IEP. Mutation of the two cysteines flanking the YADD catalytic center (C214 and C220) significantly reduced reverse transcription activity. Similar effects have been reported in retroviral reverse transcriptases such as HIV-1 and HIV-2, where cysteine mutations within the polymerase domain impair enzymatic function.^66,67^ These residues likely contribute to proper folding or positioning of the catalytic core. In contrast, mutation of the thumb-domain cysteine (C345M) led to a marked increase in reverse transcription activity relative to both the wild-type protein and other cysteine mutants. This enhancement is consistent with observations in HIV-2 RTs, where mutations near the DNA-binding domain improved both catalytic efficiency and fidelity.^67,68^ To assess any structural changes among these mutants compared to WT IEP, we generated predicted structures using AlphaFold3 and overlapped the four structures (Figure S11). Comparing these structures shows no topological changes associated with altering the activity of the cysteine mutants. Interestingly, the effect differs from that seen in non-LTR retrotransposons such as LINE-1, where mutation of a C-terminal cysteine-rich domain reduces RT activity and retromobility.^69,70^ This contrast suggests that while C-terminal cysteines may support RT function across systems, their roles can be either structurally stabilizing or regulatory, depending on context. Together, these findings suggest that the conserved thumb-domain cysteine in *Ta.it.*I1 IEP may function as a regulatory element, potentially acting as a brake on polymerization under native conditions. In contrast, the cysteines flanking the YADD motif contribute to catalytic stabilization. This modularity offers a promising strategy for engineering reverse transcriptases with improved activity or fidelity. Simple cysteine-to-methionine substitutions, particularly in the C-terminal region, may enhance RT function for biotechnological applications.

After splicing, the IEP remains bound to the intron lariat, forming a ribonucleoprotein complex that serves as the active intermediate for retromobility. During this process, the IEP stabilizes the intron and promotes the conformational changes required for the two sequential reverse splicing steps. Following insertion of the intron into the DNA target, the IEP then reverse transcribes the intron RNA into cDNA, completing the retromobility pathway.^15,41,42^ We and others have shown that the second reverse splicing step is closely linked to activation of the IEP’s reverse transcriptase domain.^52–54^ Without proper engagement of the RT domain between the first and second reverse splice steps, integration stalls after the first step, resulting in accumulation of a 3′ lariat–DNA intermediate.^52–54^ Despite the reduced reverse transcription activity observed for the C214M and C220M mutations, all *Ta.it*.I1 IEP cysteine mutants supported completion of the second reverse splicing step at levels comparable to the wild-type protein. The cysteines flanking the YADD catalytic motif may help stabilize the conformation of the RT domain. Loss of these cysteines likely impairs proper assembly or positioning of the catalytic core, thereby reducing RT efficiency. However, this disruption does not completely destabilize the RT domain, allowing sufficient engagement to support the second reverse splicing step.

The standard RT activity assay measured activity using a short poly­(rA)/oligo­(dT) template that lacks the complex secondary structures characteristic of group II introns. To address this limitation and determine whether the differential RT activities observed in the mutants similarly affects cDNA synthesis on a more complex template, we measured full-length intron cDNA synthesis following integration of C345M and C214,220M. Compared to WT IEP, C345M synthesize intron cDNA with higher processivity, producing more full-length product. However, this increase did not reach the 2-fold enhancement observed in the RT assay. While the C214,220M double mutant supported complete intron integration, C214,220M stalled after the second reverse splicing step and was unable to efficiently synthesize full-length cDNA of the intron. This further supports that the cysteines flanking the YADD motif are essential for proper RT domain stabilization and RT efficiency. Further experimentation is needed to fully understand the impact that the mutations have on processivity and template fidelity.

Following characterization of the IEP cysteine mutants, we demonstrated that maleimide-labeled IEPs retain full splicing functionality. The primary high-affinity binding site between the IEP and intron RNA lies within DIV, which encodes the IEP open reading frame. To test labeling compatibility at a potential contact site, we engineered and purified an E271C mutant. This variant, located in a region predicted to interact with DIV, exhibited splicing activity comparable to both the wild-type IEP and the C220,345M double mutant. Labeling of the E271C mutant was more efficient, suggesting that a more solvent-exposed region of the IEP is more readily labeled than a region located within the protein core. These results confirm that maleimide labeling of the *Ta.it.*I1 IEP does not interfere with its ability to promote intron splicing and lariat formation. To further evaluate the utility of labeled proteins, we tested the ability of fluorescently labeled *Ta.it.*I1 IEPs to associate with fluorescently labeled intron RNA under native conditions. Labeled RNPs formed successfully, demonstrating that site-specific labeling preserves the capacity for RNP assembly. We also show through Coomassie stain of the native PAGE that the fluorescently labeled protein does not degrade under native conditions and maintains functionality. This labeling strategy provides a highly modular and minimally perturbative approach to investigate group II intron RNP dynamics *in vitro*. Compared to global labeling or fusion-tag methods, the cysteine-based strategy offers precise spatial control and is compatible with time-resolved or single-molecule fluorescence techniques. Importantly, the platform can be readily adapted to other group II intron systems or to alternative conjugation chemistries (e.g., click-labeling or FRET pairs), supporting a broad range of applications. These include dissecting sequential assembly steps and probing RNA folding transitions. As such, this approach expands the toolkit for studying group II intron RNA–protein interactions, catalytic RNA function, and RNP-mediated mobility in real time.

## Conclusions

In this study, we show that cysteine residues are not essential for the catalytic functions of the *Ta.it.*I1 IEP. Cysteine-free IEP variants maintained lariat splicing activity and supported both steps of reverse splicing during DNA integration. Mutation of the thumb-domain cysteine enhanced reverse transcription activity, suggesting that alterations at this site may improve enzyme-DNA interactions. Furthermore, fluorescently labeled IEP variants remained active in splicing assays, underscoring the protein’s tolerance to modification. Together, these findings establish cysteine-free IEPs as useful tools for probing intron RNP assembly and function, and they provide a foundation for mechanistic and applied studies of group II intron mobility.

## Supplementary Material



## Notes

Uncropped gel images used in the figures, cysteine-to-serine *Ta.it.*I1 purification and splicing activity, purification and splicing activity of cysteine-mutated *T.el* IEP, superimposed Alphafold3 predictions of WT, C345M, C220,345M, and C214,220M IEP mutants, and protein and RNA sequences (PDF)
