# Supplementary material for: Cysteine Mutagenesis of a Group II Intron-Encoded Protein Supports Splicing, Mobility, and Site-Specific Labeling
Source: Biochemistry. 2025 Aug 29;64(21):4374–86. doi: 10.1021/acs.biochem.5c00382 (PMC12590461; doi:10.1021/acs.biochem.5c00382)
Supplement: Supplementary file 1 [file bi5c00382_si_001.pdf]

Supplementary Information

Cysteine Mutagenesis of a Group II Intron-Encoded Protein Supports Splicing, Mobility, and Site-Specific Labeling

Jasmine A. Harper[1], Sarah A. Starcovic[1], Neil Billington[1], and Aaron R. Robart*[1]

1. Department of Biochemistry and Molecular Medicine, West Virginia University, Morgantown, West Virginia, U.S.A. 26506

*Correspondence should be addressed to A.R.R.; aaron.robart@hsc.wvu.edu

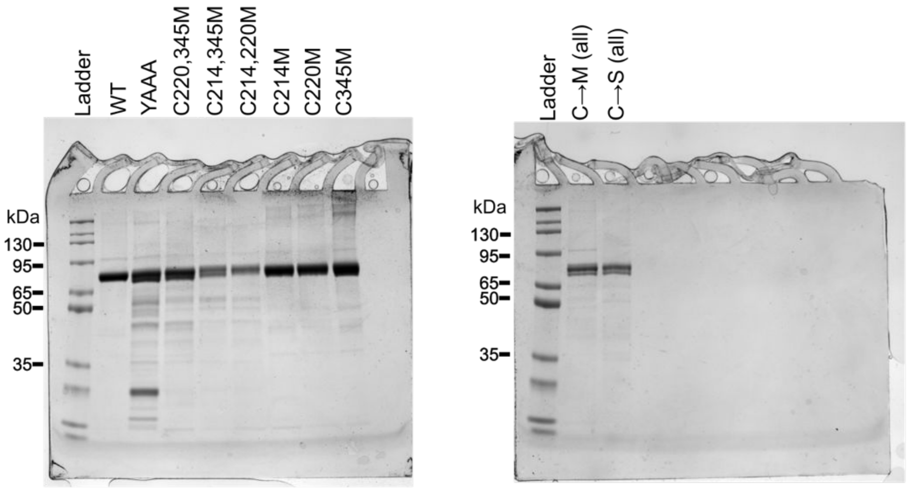

Supplementary Figure 1: Uncropped SDS-PAGE images of purified *Ta.it.*I1 IEP mutants in Figure 2B.

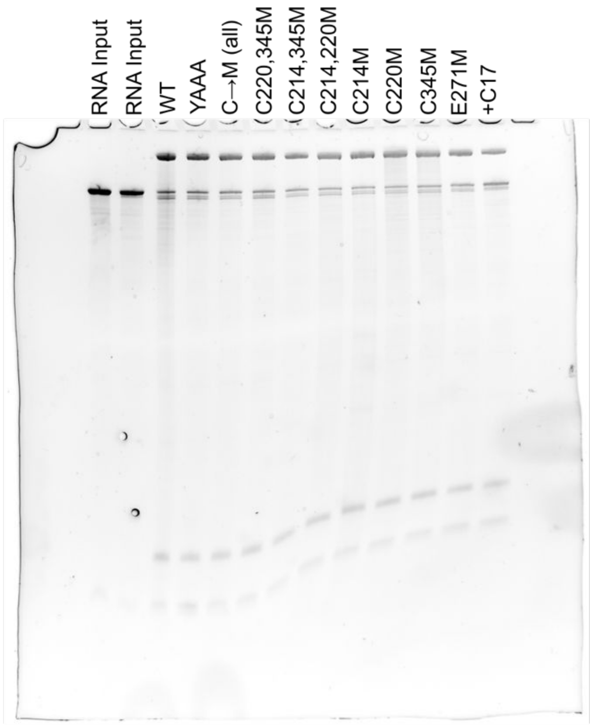

Supplementary Figure 2: Uncropped image Figure 2D. The last two lanes on the right are additional IEP mutants purified and tested for splice activity.

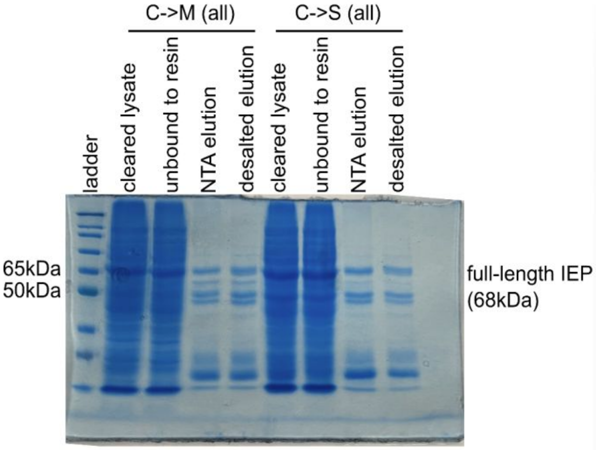

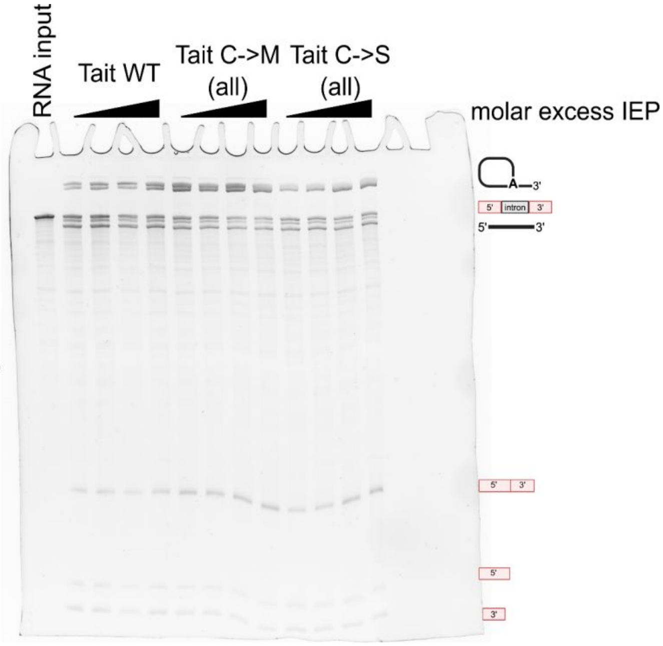

Supplementary Figure 3: Purification (left) and *in vitro* splice activity assay (right) of *Ta.it.*I1 mutants C→M (all) and C→S (all).

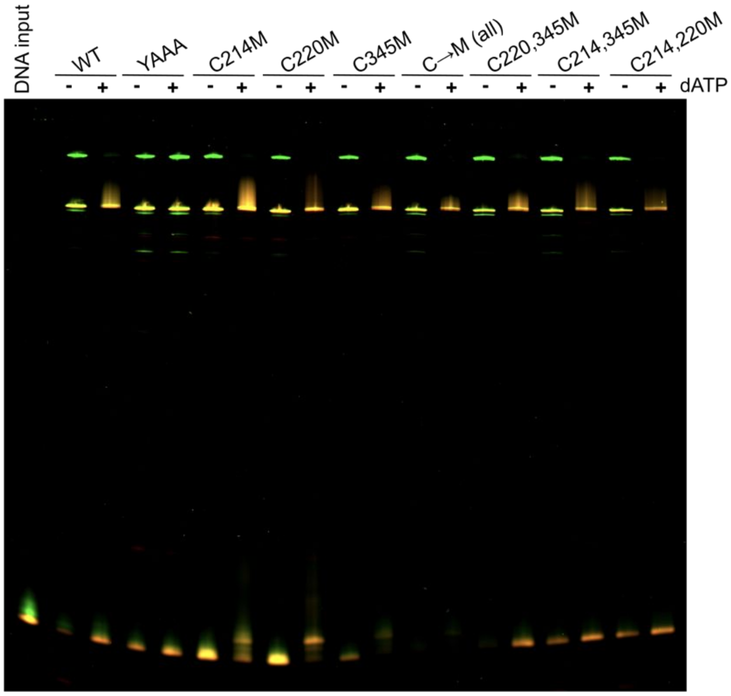

Supplementary Figure 4: Uncropped image of Figure 4B.

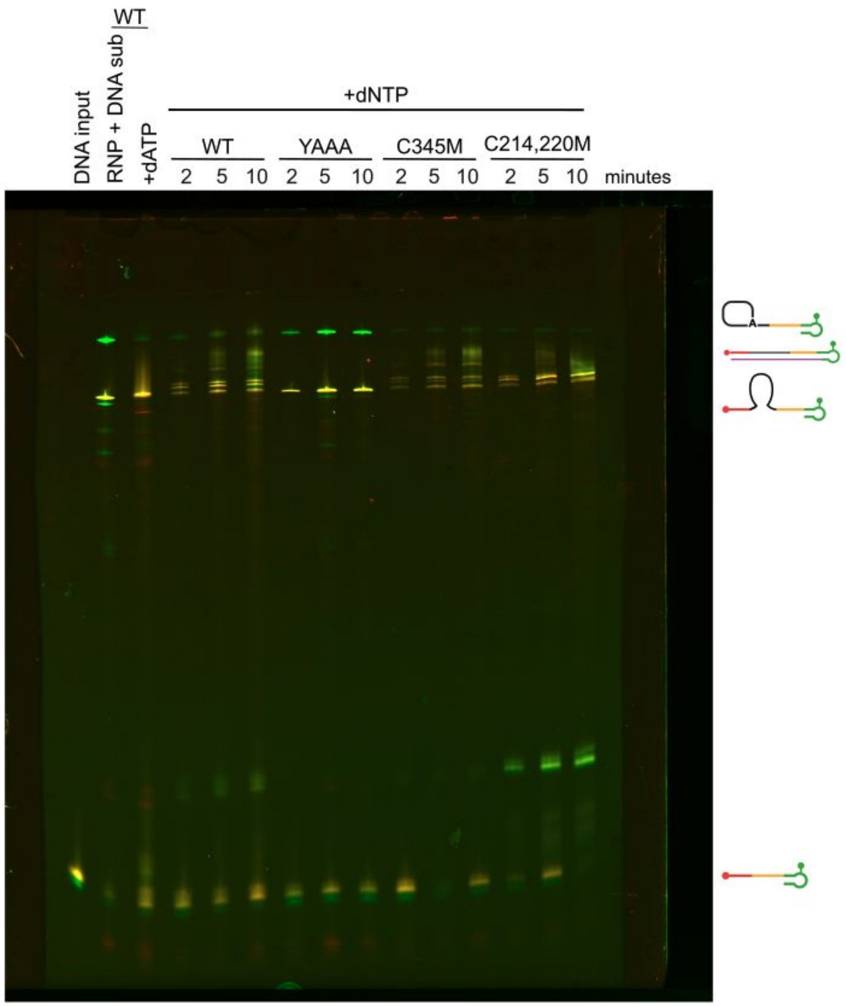

Supplementary Figure 5: Uncropped image of Figure 4D.

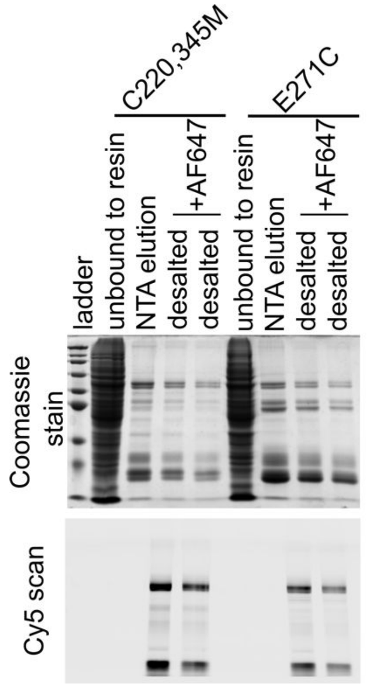

Supplementary Figure 6: Uncropped image of Figure 5B.

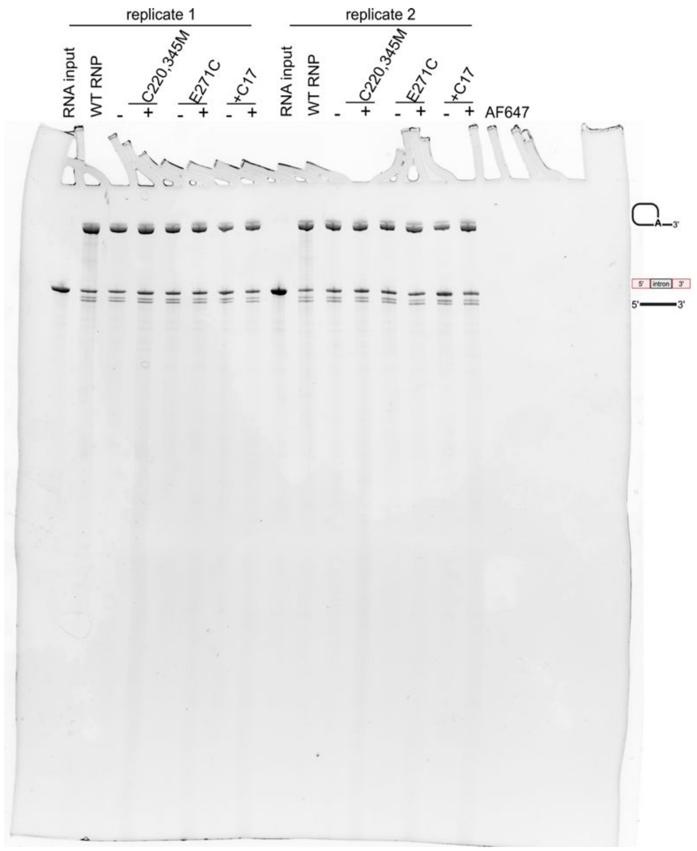

Supplementary Figure 7: Uncropped image of Figure 5C. Exons are not present because the gel was run for longer to separate out lariat from unreacted precursor RNA and hydrolytically-spliced RNA for better quantitation.

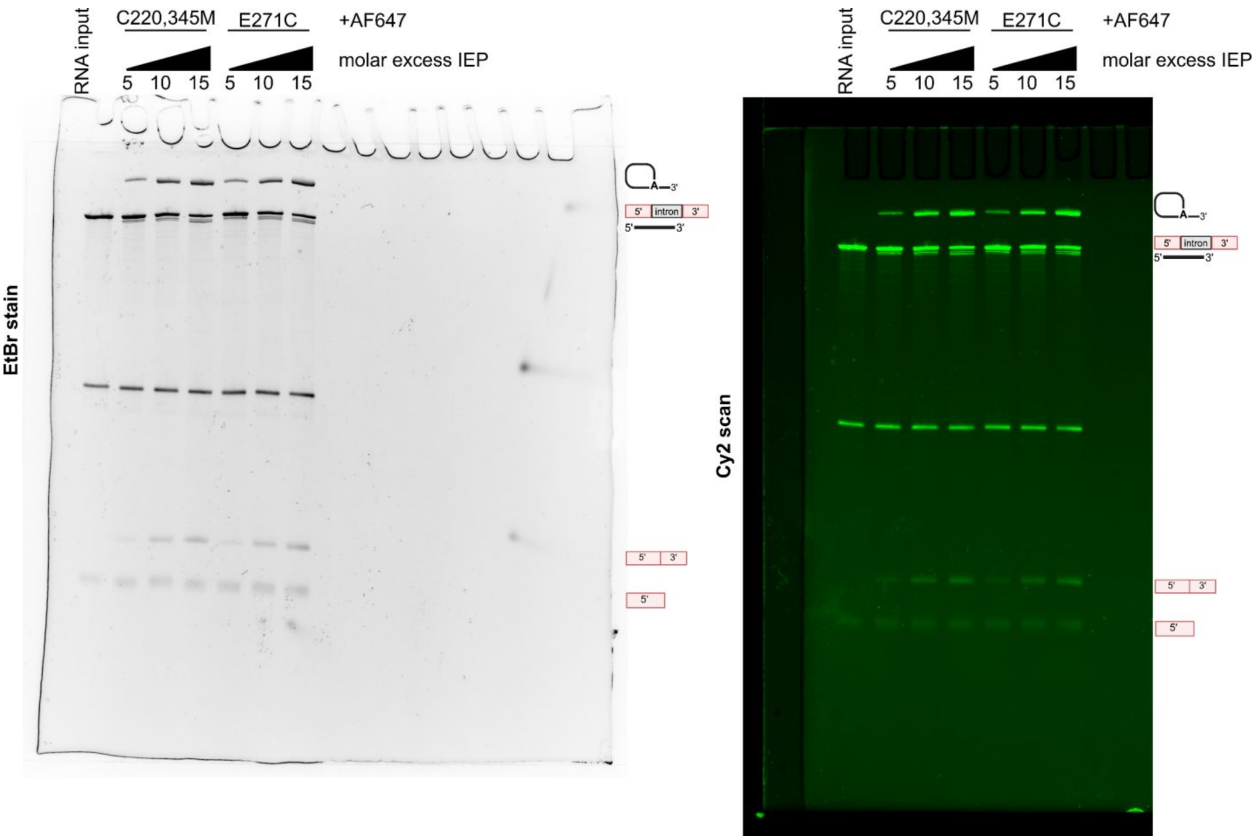

Supplementary Figure 8: Uncropped images of Figure 6B.

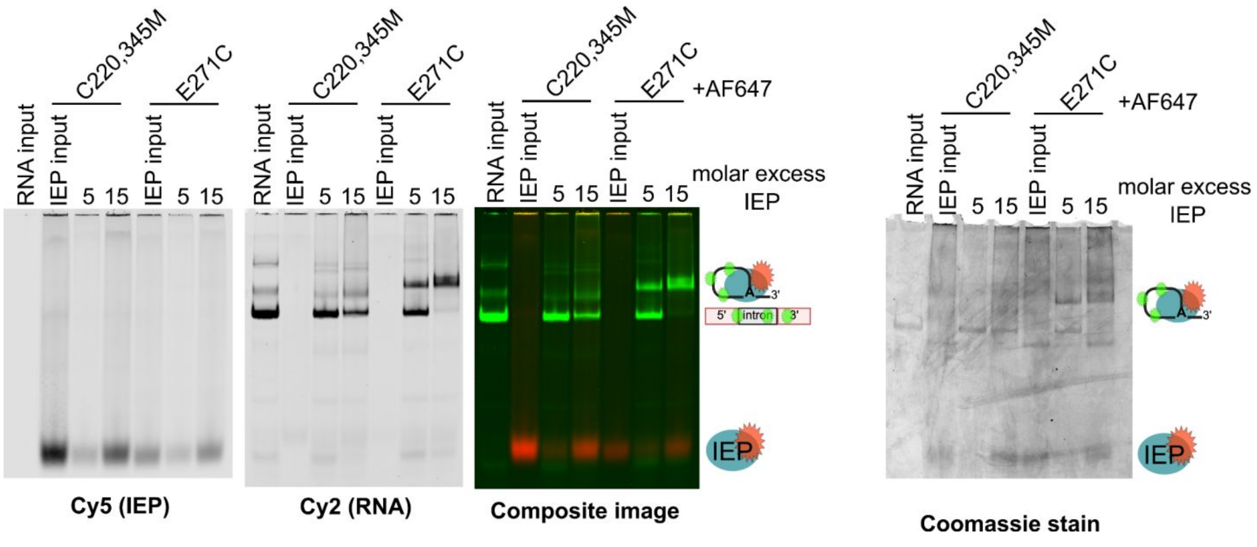

Supplementary Figure 9: Uncropped images of Figure 6C (left) and Coomassie-stained image of the same native PAGE (right) depicting the location of the AF647-labeled IEPs in native conditions.

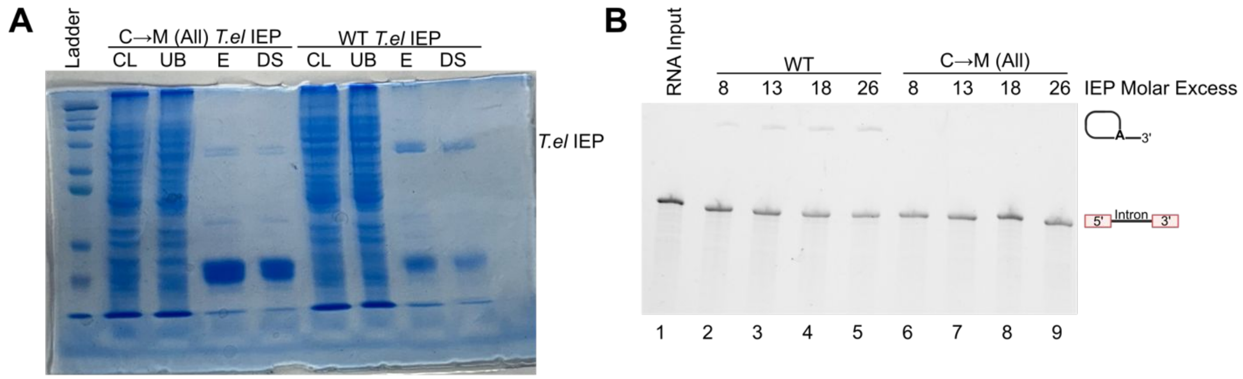

Supplementary Figure 10: Purification and *in vitro* splice activity of WT and cysteine-mutated *T.el* group II IEP. All seven endogenous cysteines in the *T.el* IEP were substituted with methionine. A) SDS-PAGE of clear lysate (CL), unbound fraction (UB), elution (E), and desalt (DS) samples taken during purification of C→M (All) and WT *T.el* group II IEP. B) 4% PAGE analysis of splicing activity of WT *T.el* group II intron with C→M (All) *T.el* IEP compared to WT *T.el* IEP.

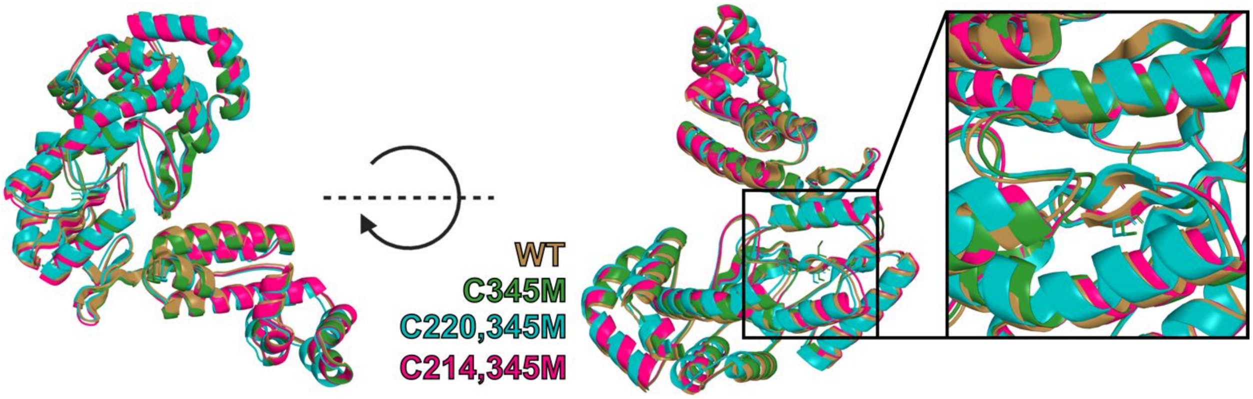

Supplementary Figure 11: Superimposed Alphafold3 predictions of WT (gold), C345M (green), C220,345M (cyan), and C214,345M (pink) *Ta.it.*I1 IEPs. The first representation on the left is the same orientation of the IEP in Figure 1A. This is then rotated 180° around the center axis to visualize the cysteines flanking the active site in the palm (black box). This area is then zoomed in to show the cysteine residues as sticks, where no major topological differences occur among the mutants compared to WT IEP.

Supplementary Table 1: List of all protein sequences used in this study

Construct Name	Description	Nucleotide sequence	Amino acid sequence
His-SUMO-TaitRT	Wild-type *Ta.it.*I1 IEP	CATATGTCAGGCCATC<br>ACCATCACCATCACCA<br>TCATGGTGGAGGGAG<br>TGGCTCGTCTGGCGG<br>GGGTTCTGGAATGTCA<br>GACTCGGAGGTGAAC<br>CAGGAAGCGAAGCCG<br>GAAGTTAAGCCGGAA<br>GTCAAGCCTGAGACG<br>CACATTAATCTGAAAG<br>TAAGTGATGGTTCCTC<br>TGAGATCTTTTTTAAGA<br>TTAAAAAAACCACCCC<br>TCTTCGTCGTTTGATG<br>GAGGCATTTGCCAAAC<br>GTCAAGGGAAGGAAAT<br>GGACTCATTGCGTTTC<br>CTTTATGATGGTATCC<br>GCATTCAGGCAGATCA<br>AACCCCGGAAGATTTG<br>GACATGGAGGACAAT<br>GATATTATCGAGGCGC<br>ATCGCGAACAAATCGG<br>TGGCGCGGCCGCGGA<br>AATGGACAGCAAGGAT<br>ATGCAACGTTTACAAA<br>CTACTCAACAGCGCG<br>GTTACCCTCTGAATCG<br>CGAGATGGAGTTCCAA<br>AAGACCACTGAGGTAC<br>ACTCTATTTCCTCCGC<br>TTCCGAAGATGGGCGT<br>AACGAAGTACAGCGCT<br>ACACGGGTAAGATGCT<br>TGAGATGATCGTCGAA<br>CGTGGAAATATGGAG<br>GCAGCTTACAAGCGTG<br>TTGTTGCAAATAAAGG	MSGHHHHHHHHGGG<br>SGSSGGGSGMSDSEV<br>NQEAKPEVKPEVKPET<br>HINLKVSDGSSEIFFKIK<br>KTTPLRRLMEAFAKRQ<br>GKEMDSLRFLYDGIRI<br>QADQTPEDLDMEDNDI<br>IEAHREQIGGAAAEMD<br>SKDMQRLQTTQQRGY<br>PLNREMEFQKTTEVHS<br>ISSASEDGRNEVQRYT<br>GKMLEMIVERGNMEA<br>AYKRVVANKGSHGVD<br>GMGVDELLPYLKENW<br>ATIKQQLLEGKYKPQP<br>VRRVEIPKPDGGKRLL<br>GIPTVLDRLIQQAIAQIL<br>NKVYNHTFSDSSYGFR<br>PGRSAKDAIKAAEAYIN<br>EGYTWVVDMDLEKFF<br>DRVNHDIIMSKLEKRIG<br>DKRVLKLIRRYLESGV<br>MINGIKVSTEEGTPQG<br>GPLSPLLANIMLDELDK<br>ELEKRGHKFCRYADD<br>CNIYVRSRSAGNRVMK<br>SIKKFIESKLKLKVNEA<br>KSAVDRPWRRKFLGF<br>SFYTKENEVRIRIHEKSI<br>KRFKEKVREITNRNKGI<br>SMENRIKRLNQITTGW<br>VNYFGLADAKSIMKTL<br>DEWIRRRLRACIWKQ<br>WKKIKTKHDNLVKLGV<br>EEQKAWEYANTRKGY<br>WRISNSPILNKTLTNKY<br>FESIGYKSLSQRYLIVH<br>NS

		GTCCCACGGGGTAGA<br>CGGTATGGGCGTCGA<br>TGAATTGCTGCCGTAC<br>TTAAAAGAAAATTGGG<br>CCACAATCAAACAACA<br>GTTGTTAGAAGGCAAG<br>TACAAACCCCAACCCG<br>TCCGTCGTGTTGAAAT<br>CCCTAAGCCAGATGGA<br>GGAAAACGTCTTCTGG<br>GGATTCCAACCGTATT<br>AGATCGTCTTATTCAA<br>CAGGCAATTGCACAGA<br>TTTTGAATAAGGTATAT<br>AATCATACTTTTTCCGA<br>CAGTTCCTATGGTTTT<br>CGTCCCGGGCGCTCT<br>GCGAAAGACGCTATTA<br>AGGCTGCTGAGGCCT<br>ATATTAACGAGGGCTA<br>TACCTGGGTCGTGGAT<br>ATGGACTTGGAGAAGT<br>TCTTTGATCGCGTGAA<br>TCACGACATTATTATG<br>TCCAAGCTTGAAAAGC<br>GCATCGGCGATAAGC<br>GTGTTCTTAAATTGATT<br>CGCCGCTATTTGGAGT<br>CCGGAGTAATGATCAA<br>TGGCATCAAGGTTAGC<br>ACTGAAGAAGGTACAC<br>CACAAGGAGGCCCAC<br>TTAGCCCTCTGTTAGC<br>TAATATTATGTTGGAT<br>GAGCTGGACAAAGAG<br>CTTGAGAAACGCGGTC<br>ACAAGTTCTGTCGTTA<br>CGCGGATGACTGCAAT<br>ATCTATGTGCGCAGTC<br>GCTCGGCTGGTAACC<br>GCGTCATGAAAAGCAT<br>TAAGAAGTTCATCGAA<br>AGTAAATTAAAGCTTA	

		AGGTAAATGAAGCGAA<br>ATCGGCGGTGGACCG<br>TCCGTGGCGTCGTAA<br>GTTCCTGGGTTTCAGT<br>TTTTATACGAAAGAGA<br>ATGAAGTACGCATTCG<br>TATCCACGAGAAATCC<br>ATCAAACGTTTTAAGG<br>AAAAAGTCCGTGAGAT<br>TACGAACCGTAACAAG<br>GGAATTAGCATGGAAA<br>ACCGCATTAAGCGCCT<br>TAATCAAATTACTACTG<br>GCTGGGTAAATTACTT<br>TGGATTAGCGGACGC<br>CAAGTCAATTATGAAA<br>ACACTGGATGAATGGA<br>TTCGTCGTCGTTTGCG<br>TGCCTGCATTTGGAAG<br>CAATGGAAAAAGATCA<br>AAACGAAACACGACAA<br>CTTGGTAAAGCTTGGA<br>GTGGAGGAACAAAAG<br>GCGTGGGAATATGCC<br>AACACACGTAAAGGTT<br>ACTGGCGTATCTCCAA<br>CTCACCAATTCTTAATA<br>AGACCCTTACAAATAA<br>ATACTTCGAATCAATT<br>GGGTACAAGTCACTGT<br>CCCAACGCTATTTAAT<br>TGTTCATAATTCGTAAT<br>AGGGATCC	
His-SUMO-TaitRTC1	C220,3455M<br>*Ta.it.*I1 IEP<br>mutant	CATATGTCACATCACC<br>ACCATCACCACCACCA<br>TGGAGGCGGTTCAGG<br>CTCTTCCGGTGGTGG<br>CTCCGACAGCGAAGT<br>GAACCAAGAAGCCAA<br>GCCGGAAGTTAAGCC<br>GGAAGTGAAGCCGGA<br>GACACATATCAACCTG<br>AAGGTGTCCGATGGTT<br>CCAGCGAGATTTTTTT	HMSHHHHHHHHGGG<br>SGSSGGGSDSEVNQE<br>AKPEVKPEVKPETHINL<br>KVSDGSSEIFFKIKKTT<br>PLRRLMEAFAKRQGKE<br>MDSLRFLYDGIRIQAD<br>QTPEDLDMEDNDIIEA<br>HREQIGGTDSKDMQR<br>LQTTQQRGYPLNREM<br>EFQKTTEVHSISSASE<br>DGRNEVQRYTGKMLE

		CAAGATCAAGAAAACG<br>ACCCCGCTGCGTCGG<br>CTGATGGAGGCGTTTG<br>CAAAGCGCCAGGGTA<br>AAGAAATGGATTCTCT<br>GCGTTTCCTGTATGAT<br>GGCATCCGTATTCAAG<br>CAGACCAGACGCCAG<br>AGGATCTGGATATGGA<br>AGACAACGATATTATT<br>GAAGCGCATCGAGAG<br>CAAATCGGTGGTACCG<br>ATAGTAAGGACATGCA<br>GAGATTACAGACCACC<br>CAGCAGCGTGGTTAC<br>CCGTTGAATAGAGAAA<br>TGGAATTCCAGAAAAC<br>TACGGAGGTGCACAG<br>CATCAGCTCCGCGAG<br>CGAAGACGGTAGGAA<br>CGAGGTCCAGAGGTA<br>CACGGGCAAGATGTT<br>GGAGATGATTGTGGAA<br>CGTGGCAACATGGAA<br>GCTGCCTATAAGCGC<br>GTGGTTGCAAACAAGG<br>GCTCGCACGGTGTGG<br>ATGGTATGGGCGTTGA<br>CGAGTTGCTGCCGTAT<br>TTGAAGGAGAACTGG<br>GCAACCATTAAACAAC<br>AACTGTTAGAGGGTAA<br>ATACAAGCCGCAACCG<br>GTGCGTAGAGTTGAGA<br>TCCCGAAACCGGATG<br>GTGGCAAACGCCTTCT<br>GGGAATCCCGACCGT<br>TCTGGACCGCTTGATC<br>CAACAAGCTATCGCCC<br>AGATTCTGAATAAAGT<br>GTATAACCACACCTTT<br>TCGGACTCCAGCTATG<br>GCTTTCGTCCAGGTCG<br>TTCTGCGAAAGATGCT<br>ATTAAGGCTGCGGAAG<br>CGTACATTAACGAGGG<br>TTATACCTGGGTTGTT<br>GATATGGACCTGGAGA<br>AGTTCTTCGATCGTGT<br>CAATCACGACATTATC	MIVERGNMEAAYKRVV<br>ANKGSHGVDGMGVDE<br>LLPYLKENWATIKQQLL<br>EGKYKPQPVRRVEIPK<br>PDGGKRLLGIPTVLDR<br>LIQQAIAQILNKVYNHT<br>FSDSSYGFRPGRSAK<br>DAIKAAEAYINEGYTW<br>VVDMDLEKFFDRVNH<br>DIIMSKLEKRIGDKRVL<br>KLIRRYLESGVMINGIK<br>VSTEEGTPQGGPLSPL<br>LANIMLDELDKELEKR<br>GHKFCRYADDMNIYVR<br>SRSAGNRVMKSIKKFIE<br>SKLKLKVNEAKSAVDR<br>PWRRKFLGFSFYTKEN<br>EVRIRIHEKSIKRFKEK<br>VREITNRNKGISMENRI<br>KRLNQITTGWVNYFGL<br>ADAKSIMKTLDEWIRR<br>RLRAMIWKQWKKIKTK<br>HDNLVKLGVEEQKAW<br>EYANTRKGYWRISNSP<br>ILNKTLTNKYFESIGYK<br>SLSQRYLIVHNS

		ATGAGCAAACTGGAGA AACGCATCGGTGATAA ACGTGTACTCAAGTTG ATCCGTCGTTACTTGG AGAGCGGCGTGATGA TTAACGGTATCAAGGT CAGCACCGAAGAGGG CACTCCGCAGGGCGG CCCCCTGAGCCCGCT GTTGGCGAACATTATG CTGGACGAGCTGGAC AAAGAGCTGGAGAAG CGTGGTCATAAATTCT GTCGTTACGCTGACGA CATGAATATTTATGTTC GTAGCCGTTCTGCGG GTAACCGTGTTATGAA AAGCATCAAAAAATTC ATCGAGAGCAAACTGA AGCTGAAAGTTAATGA AGCGAAGTCGGCGGT GGACCGTCCGTGGCG CCGTAAGTTCCTTGGT TTTAGCTTTTATACCAA AGAGAACGAAGTTCGT ATCCGTATTCACGAAA AGAGCATCAAACGCTT TAAGGAAAAGGTTCGC GAAATCACCAATCGTA ATAAGGGGATCAGCAT GGAAAACCGTATCAAG CGCCTCAACCAGATCA CCACCGGCTGGGTCA ACTATTTTGGTCTGGC GGACGCGAAATCTATT ATGAAAACGCTAGACG AATGGATTCGCCGTCG CTTACGTGCAATGATT TGGAAACAATGGAAAA AGATTAAGACCAAACA TGATAATCTGGTTAAA CTGGGTGTGGAGGAA CAGAAAGCATGGGAAT ACGCCAATACGCGCAA AGGTTACTGGCGTATC AGCAATAGCCCGATTC TGAACAAGACCTTGAC CAACAAGTACTTCGAG TCGATCGGCTACAAAA GCTTGTCTCAGCGCTA	

		CCTGATCGTCCACAAC<br>AGCTAATAGGATCC	
His-SUMO-TaitRTC2	C214,345M<br>*Ta.it.*I1 IEP<br>mutant	CATATGTCACATCACC<br>ACCATCACCACCACCA<br>TGGAGGCGGTTCAGG<br>CTCTTCCGGTGGTGG<br>CTCCGACAGCGAAGT<br>GAACCAAGAAGCCAA<br>GCCGGAAGTTAAGCC<br>GGAAGTGAAGCCGGA<br>GACACATATCAACCTG<br>AAGGTGTCCGATGGTT<br>CCAGCGAGATTTTTTT<br>CAAGATCAAGAAAACG<br>ACCCCGCTGCGTCGG<br>CTGATGGAGGCGTTTG<br>CAAAGCGCCAGGGTA<br>AAGAAATGGATTCTCT<br>GCGTTTCCTGTATGAT<br>GGCATCCGTATTCAAG<br>CAGACCAGACGCCAG<br>AGGATCTGGATATGGA<br>AGACAACGATATTATT<br>GAAGCGCATCGAGAG<br>CAAATCGGTGGTACCG<br>ATAGTAAGGACATGCA<br>GAGATTACAGACCACC<br>CAGCAGCGTGGTTAC<br>CCGTTGAATAGAGAAA<br>TGGAATTCCAGAAAAC<br>TACGGAGGTGCACAG<br>CATCAGCTCCGCGAG<br>CGAAGACGGTAGGAA<br>CGAGGTCCAGAGGTA<br>CACGGGCAAGATGTT<br>GGAGATGATTGTGGAA<br>CGTGGCAACATGGAA<br>GCTGCCTATAAGCGC<br>GTGGTTGCAAACAAGG<br>GCTCGCACGGTGTGG<br>ATGGTATGGGCGTTGA<br>CGAGTTGCTGCCGTAT<br>TTGAAGGAGAACTGG<br>GCAACCATTAAACAAC<br>AACTGTTAGAGGGTAA<br>ATACAAGCCGCAACCG<br>GTGCGTAGAGTTGAGA<br>TCCCGAAACCGGATG<br>GTGGCAAACGCCTTCT	HMSHHHHHHHHGGG<br>SGSSGGGSDSEVNQE<br>AKPEVKPEVKPETHINL<br>KVSDGSSEIFFKIKKTT<br>PLRRLMEAFAKRQGKE<br>MDSLRFLYDGIRIQAD<br>QTPEDLDMEDNDIIEA<br>HREQIGGTDSKDMQR<br>LQTTQQRGYPLNREM<br>EFQKTTEVHSISSASE<br>DGRNEVQRYTGKMLE<br>MIVERGNMEAAYKRVV<br>ANKGSHGVDGMGVDE<br>LLPYLKENWATIKQQLL<br>EGKYKPQPVRRVEIPK<br>PDGGKRLLGIPTVLDR<br>LIQQAIAQILNKVYNHT<br>FSDSSYGFRPGRSAK<br>DAIKAAEAYINEGYTW<br>VVDMDLEKFFDRVNH<br>DIIMSKLEKRIGDKRVL<br>KLIRRYLESGVMINGIK<br>VSTEEGTPQGGPLSPL<br>LANIMLDELDKELEKR<br>GHKFMRYADDCNIYVR<br>SRSAGNRVMKSIKKFIE<br>SKLKLKVNEAKSAVDR<br>PWRRKFLGFSFYTKEN<br>EVRIRIHEKSIKRFKEK<br>VREITNRNKGISMENRI<br>KRLNQITTGWVNYFGL<br>ADAKSIMKTLDEWIRR<br>RLRAMIWKQWKKIKTK<br>HDNLVKLGVEEQKAW<br>EYANTRKGYWRISNSP<br>ILNKTLTNKYFESIGYK<br>SLSQRYLIVHNS

		GGGAATCCCGACCGT<br>TCTGGACCGCTTGATC<br>CAACAAGCTATCGCCC<br>AGATTCTGAATAAAGT<br>GTATAACCACACCTTT<br>TCGGACTCCAGCTATG<br>GCTTTCGTCCAGGTCG<br>TTCTGCGAAAGATGCT<br>ATTAAGGCTGCGGAAG<br>CGTACATTAACGAGGG<br>TTATACCTGGGTTGTT<br>GATATGGACCTGGAGA<br>AGTTCTTCGATCGTGT<br>CAATCACGACATTATC<br>ATGAGCAAACTGGAGA<br>AACGCATCGGTGATAA<br>ACGTGTACTCAAGTTG<br>ATCCGTCGTTACTTGG<br>AGAGCGGCGTGATGA<br>TTAACGGTATCAAGGT<br>CAGCACCGAAGAGGG<br>CACTCCGCAGGGCGG<br>CCCCCTGAGCCCGCT<br>GTTGGCGAACATTATG<br>CTGGACGAGCTGGAC<br>AAAGAGCTGGAGAAG<br>CGTGGTCATAAATTCA<br>TGCGTTACGCTGACGA<br>CTGTAATATTTATGTTC<br>GTAGCCGTTCTGCGG<br>GTAACCGTGTTATGAA<br>AAGCATCAAAAAATTC<br>ATCGAGAGCAAACTGA<br>AGCTGAAAGTTAATGA<br>AGCGAAGTCGGCGGT<br>GGACCGTCCGTGGCG<br>CCGTAAGTTCCTTGGT<br>TTTAGCTTTTATACCAA<br>AGAGAACGAAGTTCGT<br>ATCCGTATTCACGAAA<br>AGAGCATCAAACGCTT<br>TAAGGAAAAGGTTCGC<br>GAAATCACCAATCGTA<br>ATAAGGGGATCAGCAT<br>GGAAAACCGTATCAAG<br>CGCCTCAACCAGATCA<br>CCACCGGCTGGGTCA<br>ACTATTTTGGTCTGGC<br>GGACGCGAAATCTATT<br>ATGAAAACGCTAGACG	

		AATGGATTCGCCGTCG CTTACGTGCAATGATT TGGAAACAATGGAAAA AGATTAAGACCAAACA TGATAATCTGGTTAAA CTGGGTGTGGAGGAA CAGAAAGCATGGGAAT ACGCCAATACGCGCAA AGGTTACTGGCGTATC AGCAATAGCCCGATTC TGAACAAGACCTTGAC CAACAAGTACTTCGAG TCGATCGGCTACAAAA GCTTGTCTCAGCGCTA CCTGATCGTCCACAAC AGCTAATAGGATCC	
His-SUMO-TaitRTC3	C214,220M *Ta.it.*I1 IEP mutant	CATATGTCACATCACC ACCATCACCACCACCA TGGAGGCGGTTCAGG CTCTTCCGGTGGTGG CTCCGACAGCGAAGT GAACCAAGAAGCCAA GCCGGAAGTTAAGCC GGAAGTGAAGCCGGA GACACATATCAACCTG AAGGTGTCCGATGGTT CCAGCGAGATTTTTTT CAAGATCAAGAAAACG ACCCCGCTGCGTCGG CTGATGGAGGCGTTTG CAAAGCGCCAGGGTA AAGAAATGGATTCTCT GCGTTTCCTGTATGAT GGCATCCGTATTCAAG CAGACCAGACGCCAG AGGATCTGGATATGGA AGACAACGATATTATT GAAGCGCATCGAGAG CAAATCGGTGGTACCG ATAGTAAGGACATGCA GAGATTACAGACCACC CAGCAGCGTGGTTAC CCGTTGAATAGAGAAA TGGAATTCCAGAAAAC TACGGAGGTGCACAG CATCAGCTCCGCGAG CGAAGACGGTAGGAA CGAGGTCCAGAGGTA CACGGGCAAGATGTT	HMSHHHHHHHHGGG SGSSGGGSDSEVNQE AKPEVKPEVKPETHINL KVSDGSSEIFFKIKKTT PLRRLMEAFAKRQGKE MDSLRFLYDGIRIQAD QTPEDLDMEDNDIIEA HREQIGGTDSKDMQR LQTTQQRGYPLNREM EFQKTTEVHSISSASE DGRNEVQRYTGKMLE MIVERGNMEAAYKRVV ANKGSHGVDGMGVDE LLPYLKENWATIKQQLL EGKYKPQPVRRVEIPK PDGGKRLLGIPTVLDR LIQQAIAQILNKVYNHT FSDSSYGFRPGRSAK DAIKAAEAYINEGYTW VVDMDLEKFFDRVNH DIIMSKLEKRIGDKRVL KLIRRYLESGVMINGIK VSTEEGTPQGGPLSPL LANIMLDELDKELEKR GHKFMRYADDMNIYV RSRSAGNRVMKSIKKF IESKLKLKVNEAKSAVD RPWRRKFLGFSFYTKE NEVRIRIHEKSIKRFKE KVREITNRNKGISMEN RIKRLNQITTGWVNYF GLADAKSIMKTLDEWI RRRLRACIWKQWKKIK

		GGAGATGATTGTGGAA<br>CGTGGCAACATGGAA<br>GCTGCCTATAAGCGC<br>GTGGTTGCAAACAAGG<br>GCTCGCACGGTGTGG<br>ATGGTATGGGCGTTGA<br>CGAGTTGCTGCCGTAT<br>TTGAAGGAGAACTGG<br>GCAACCATTAAACAAC<br>AACTGTTAGAGGGTAA<br>ATACAAGCCGCAACCG<br>GTGCGTAGAGTTGAGA<br>TCCCGAAACCGGATG<br>GTGGCAAACGCCTTCT<br>GGGAATCCCGACCGT<br>TCTGGACCGCTTGATC<br>CAACAAGCTATCGCCC<br>AGATTCTGAATAAAGT<br>GTATAACCACACCTTT<br>TCGGACTCCAGCTATG<br>GCTTTCGTCCAGGTCG<br>TTCTGCGAAAGATGCT<br>ATTAAGGCTGCGGAAG<br>CGTACATTAACGAGGG<br>TTATACCTGGGTTGTT<br>GATATGGACCTGGAGA<br>AGTTCTTCGATCGTGT<br>CAATCACGACATTATC<br>ATGAGCAAACTGGAGA<br>AACGCATCGGTGATAA<br>ACGTGTACTCAAGTTG<br>ATCCGTCGTTACTTGG<br>AGAGCGGCGTGATGA<br>TTAACGGTATCAAGGT<br>CAGCACCGAAGAGGG<br>CACTCCGCAGGGCGG<br>CCCCCTGAGCCCGCT<br>GTTGGCGAACATTATG<br>CTGGACGAGCTGGAC<br>AAAGAGCTGGAGAAG<br>CGTGGTCATAAATTCA<br>TGCGTTACGCTGACGA<br>CATGAATATTTATGTTC<br>GTAGCCGTTCTGCGG<br>GTAACCGTGTTATGAA<br>AAGCATCAAAAAATTC<br>ATCGAGAGCAAACTGA<br>AGCTGAAAGTTAATGA<br>AGCGAAGTCGGCGGT<br>GGACCGTCCGTGGCG	TKHDNLVKLGVEEQKA<br>WEYANTRKGYWRISN<br>SPILNKTLTNKYFESIG<br>YKSLSQRYLIVHNS

		CCGTAAGTTCCTTGGT<br>TTTAGCTTTTATACCAA<br>AGAGAACGAAGTTCGT<br>ATCCGTATTCACGAAA<br>AGAGCATCAAACGCTT<br>TAAGGAAAAGGTTCGC<br>GAAATCACCAATCGTA<br>ATAAGGGGATCAGCAT<br>GGAAAACCGTATCAAG<br>CGCCTCAACCAGATCA<br>CCACCGGCTGGGTCA<br>ACTATTTTGGTCTGGC<br>GGACGCGAAATCTATT<br>ATGAAAACGCTAGACG<br>AATGGATTCGCCGTCG<br>CTTACGTGCATGTATT<br>TGGAAACAATGGAAAA<br>AGATTAAGACCAAACA<br>TGATAATCTGGTTAAA<br>CTGGGTGTGGAGGAA<br>CAGAAAGCATGGGAAT<br>ACGCCAATACGCGCAA<br>AGGTTACTGGCGTATC<br>AGCAATAGCCCGATTC<br>TGAACAAGACCTTGAC<br>CAACAAGTACTTCGAG<br>TCGATCGGCTACAAAA<br>GCTTGTCTCAGCGCTA<br>CCTGATCGTCCACAAC<br>AGCTAATAGGATCC	
His-SUMO-TaitRTM1	C214M<br>*Ta.it.*I1 IEP<br>mutant	CATATGTCACATCACC<br>ACCATCACCACCACCA<br>TGGAGGCGGTTCAGG<br>CTCTTCCGGTGGTGG<br>CTCCGACAGCGAAGT<br>GAACCAAGAAGCCAA<br>GCCGGAAGTTAAGCC<br>GGAAGTGAAGCCGGA<br>GACACATATCAACCTG<br>AAGGTGTCCGATGGTT<br>CCAGCGAGATTTTTTT<br>CAAGATCAAGAAAACG<br>ACCCCGCTGCGTCGG<br>CTGATGGAGGCGTTTG<br>CAAAGCGCCAGGGTA<br>AAGAAATGGATTCTCT<br>GCGTTTCCTGTATGAT<br>GGCATCCGTATTCAAG<br>CAGACCAGACGCCAG	HMSHHHHHHHHGGG<br>SGSSGGGSDSEVNQE<br>AKPEVKPEVKPETHINL<br>KVSDGSSEIFFKIKKTT<br>PLRRLMEAFAKRQGKE<br>MDSLRFLYDGIRIQAD<br>QTPEDLDMEDNDIIEA<br>HREQIGGTDSKDMQR<br>LQTTQQRGYPLNREM<br>EFQKTTEVHSISSASE<br>DGRNEVQRYTGKMLE<br>MIVERGNMEAAYKRVV<br>ANKGSHGVDGMGVDE<br>LLPYLKENWATIKQQLL<br>EGKYKPQPVRRVEIPK<br>PDGGKRLLGIPTVLDR<br>LIQQAIAQILNKVYNHT<br>FSDSSYGFRPGRSAK<br>DAIKAAEAYINEGYTW

		AGGATCTGGATATGGA<br>AGACAACGATATTATT<br>GAAGCGCATCGAGAG<br>CAAATCGGTGGTACCG<br>ATAGTAAGGACATGCA<br>GAGATTACAGACCACC<br>CAGCAGCGTGGTTAC<br>CCGTTGAATAGAGAAA<br>TGGAATTCCAGAAAAC<br>TACGGAGGTGCACAG<br>CATCAGCTCCGCGAG<br>CGAAGACGGTAGGAA<br>CGAGGTCCAGAGGTA<br>CACGGGCAAGATGTT<br>GGAGATGATTGTGGAA<br>CGTGGCAACATGGAA<br>GCTGCCTATAAGCGC<br>GTGGTTGCAAACAAGG<br>GCTCGCACGGTGTGG<br>ATGGTATGGGCGTTGA<br>CGAGTTGCTGCCGTAT<br>TTGAAGGAGAACTGG<br>GCAACCATTAAACAAC<br>AACTGTTAGAGGGTAA<br>ATACAAGCCGCAACCG<br>GTGCGTAGAGTTGAGA<br>TCCCGAAACCGGATG<br>GTGGCAAACGCCTTCT<br>GGGAATCCCGACCGT<br>TCTGGACCGCTTGATC<br>CAACAAGCTATCGCCC<br>AGATTCTGAATAAAGT<br>GTATAACCACACCTTT<br>TCGGACTCCAGCTATG<br>GCTTTCGTCCAGGTCG<br>TTCTGCGAAAGATGCT<br>ATTAAGGCTGCGGAAG<br>CGTACATTAACGAGGG<br>TTATACCTGGGTTGTT<br>GATATGGACCTGGAGA<br>AGTTCTTCGATCGTGT<br>CAATCACGACATTATC<br>ATGAGCAAACTGGAGA<br>AACGCATCGGTGATAA<br>ACGTGTACTCAAGTTG<br>ATCCGTCGTTACTTGG<br>AGAGCGGCGTGATGA<br>TTAACGGTATCAAGGT<br>CAGCACCGAAGAGGG<br>CACTCCGCAGGGCGG	VVDMDLEKFFDRVNH<br>DIIMSKLEKRIGDKRVL<br>KLIRRYLESGVMINGIK<br>VSTEEGTPQGGPLSPL<br>LANIMLDELDKELEKR<br>GHKFMRYADDCNIYVR<br>SRSAGNRVMKSIKKFIE<br>SKLKLKVNEAKSAVDR<br>PWRRKFLGFSFYTKEN<br>EVRIRIHEKSIKRFKEK<br>VREITNRNKGISMENRI<br>KRLNQITTGWVNYFGL<br>ADAKSIMKTLDEWIRR<br>RLRACIWKQWKKIKTK<br>HDNLVKLGVEEQKAW<br>EYANTRKGYWRISNSP<br>ILNKTLTNKYFESIGYK<br>SLSQRYLIVHNS

		CCCCCTGAGCCCGCT GTTGGCGAACATTATG CTGGACGAGCTGGAC AAAGAGCTGGAGAAG CGTGGTCATAAATTCA TGCGTTACGCTGACGA CTGTAATATTTATGTTC GTAGCCGTTCTGCGG GTAACCGTGTTATGAA AAGCATCAAAAAATTC ATCGAGAGCAAACTGA AGCTGAAAGTTAATGA AGCGAAGTCGGCGGT GGACCGTCCGTGGCG CCGTAAGTTCCTTGGT TTTAGCTTTTATACCAA AGAGAACGAAGTTCGT ATCCGTATTCACGAAA AGAGCATCAAACGCTT TAAGGAAAAGGTTCGC GAAATCACCAATCGTA ATAAGGGGATCAGCAT GGAAAACCGTATCAAG CGCCTCAACCAGATCA CCACCGGCTGGGTCA ACTATTTTGGTCTGGC GGACGCGAAATCTATT ATGAAAACGCTAGACG AATGGATTCGCCGTCG CTTACGTGCATGTATT TGGAAACAATGGAAAA AGATTAAGACCAAACA TGATAATCTGGTTAAA CTGGGTGTGGAGGAA CAGAAAGCATGGGAAT ACGCCAATACGCGCAA AGGTTACTGGCGTATC AGCAATAGCCCGATTC TGAACAAGACCTTGAC CAACAAGTACTTCGAG TCGATCGGCTACAAAA GCTTGTCTCAGCGCTA CCTGATCGTCCACAAC AGCTAATAGGATCC	
His-SUMO-TaitRTM2	C220M *Ta.it.*I1 IEP mutant	CATATGTCACATCACC ACCATCACCACCACCA TGGAGGCGGTTCAGG CTCTTCCGGTGGTGG CTCCGACAGCGAAGT	HMSHHHHHHHHGGG SGSSGGGSDSEVNQE AKPEVKPEVKPETHINL KVSDGSSEIFFKIKKTT PLRRLMEAFAKRQGKE

		GAACCAAGAAGCCAA GCCGGAAGTTAAGCC GGAAGTGAAGCCGGA GACACATATCAACCTG AAGGTGTCCGATGGTT CCAGCGAGATTTTTTT CAAGATCAAGAAAACG ACCCCGCTGCGTCGG CTGATGGAGGCGTTTG CAAAGCGCCAGGGTA AAGAAATGGATTCTCT GCGTTTCCTGTATGAT GGCATCCGTATTCAAG CAGACCAGACGCCAG AGGATCTGGATATGGA AGACAACGATATTATT GAAGCGCATCGAGAG CAAATCGGTGGTACCG ATAGTAAGGACATGCA GAGATTACAGACCACC CAGCAGCGTGGTTAC CCGTTGAATAGAGAAA TGGAATTCCAGAAAAC TACGGAGGTGCACAG CATCAGCTCCGCGAG CGAAGACGGTAGGAA CGAGGTCCAGAGGTA CACGGGCAAGATGTT GGAGATGATTGTGGAA CGTGGCAACATGGAA GCTGCCTATAAGCGC GTGGTTGCAAACAAGG GCTCGCACGGTGTGG ATGGTATGGGCGTTGA CGAGTTGCTGCCGTAT TTGAAGGAGAACTGG GCAACCATTAAACAAC AACTGTTAGAGGGTAA ATACAAGCCGCAACCG GTGCGTAGAGTTGAGA TCCCGAAACCGGATG GTGGCAAACGCCTTCT GGGAATCCCGACCGT TCTGGACCGCTTGATC CAACAAGCTATCGCCC AGATTCTGAATAAAGT GTATAACCACACCTTT TCGGACTCCAGCTATG GCTTTCGTCCAGGTCG TTCTGCGAAAGATGCT	MDSLRFLYDGIRIQAD QTPEDLDMEDNDIIEA HREQIGGTDSKDMQR LQTTQQRGYPLNREM EFQKTTEVHSISSASE DGRNEVQRYTGKMLE MIVERGNMEAAYKRVV ANKGSHGVDGMGVDE LLPYLKENWATIKQQLL EGKYKPQPVRRVEIPK PDGGKRLLGIPTVLDR LIQQAIAQILNKVYNHT FSDSSYGFRPGRSAK DAIKAAEAYINEGYTW VVDMDLEKFFDRVNH DIIMSKLEKRIGDKRVL KLIRRYLESGVMINGIK VSTEEGTPQGGPLSPL LANIMLDELDKELEKR GHKFCRYADDMNIYVR SRSAGNRVMKSIKKFIE SKLKLKVNEAKSAVDR PWRRKFLGFSFYTKEN EVRIRIHEKSIKRFKEK VREITNRNKGISMENRI KRLNQITTGWVNYFGL ADAKSIMKTLDEWIRR RLRACIWKQWKKIKTK HDNLVKLGVEEQKAW EYANTRKGYWRISNSP ILNKTLTNKYFESIGYK SLSQRYLIVHNS

		ATTAAGGCTGCGGAAG CGTACATTAACGAGGG TTATACCTGGGTTGTT GATATGGACCTGGAGA AGTTCTTCGATCGTGT CAATCACGACATTATC ATGAGCAAACTGGAGA AACGCATCGGTGATAA ACGTGTACTCAAGTTG ATCCGTCGTTACTTGG AGAGCGGCGTGATGA TTAACGGTATCAAGGT CAGCACCGAAGAGGG CACTCCGCAGGGCGG CCCCCTGAGCCCGCT GTTGGCGAACATTATG CTGGACGAGCTGGAC AAAGAGCTGGAGAAG CGTGGTCATAAATTCT GTCGTTACGCTGACGA CATGAATATTTATGTTC GTAGCCGTTCTGCGG GTAACCGTGTTATGAA AAGCATCAAAAAATTC ATCGAGAGCAAACTGA AGCTGAAAGTTAATGA AGCGAAGTCGGCGGT GGACCGTCCGTGGCG CCGTAAGTTCCTTGGT TTTAGCTTTTATACCAA AGAGAACGAAGTTCGT ATCCGTATTCACGAAA AGAGCATCAAACGCTT TAAGGAAAAGGTTCGC GAAATCACCAATCGTA ATAAGGGGATCAGCAT GGAAAACCGTATCAAG CGCCTCAACCAGATCA CCACCGGCTGGGTCA ACTATTTTGGTCTGGC GGACGCGAAATCTATT ATGAAAACGCTAGACG AATGGATTCGCCGTCG CTTACGTGCATGTATT TGGAAACAATGGAAAA AGATTAAGACCAAACA TGATAATCTGGTTAAA CTGGGTGTGGAGGAA CAGAAAGCATGGGAAT ACGCCAATACGCGCAA	

		AGGTTACTGGCGTATC<br>AGCAATAGCCCGATTC<br>TGAACAAGACCTTGAC<br>CAACAAGTACTTCGAG<br>TCGATCGGCTACAAAA<br>GCTTGTCTCAGCGCTA<br>CCTGATCGTCCACAAC<br>AGCTAATAGGATCC	
His-SUMO-TaitRTM3	C345M<br>*Ta.it.*I1 IEP<br>mutant	CATATGTCACATCACC<br>ACCATCACCACCACCA<br>TGGAGGCGGTTCAGG<br>CTCTTCCGGTGGTGG<br>CTCCGACAGCGAAGT<br>GAACCAAGAAGCCAA<br>GCCGGAAGTTAAGCC<br>GGAAGTGAAGCCGGA<br>GACACATATCAACCTG<br>AAGGTGTCCGATGGTT<br>CCAGCGAGATTTTTTT<br>CAAGATCAAGAAAACG<br>ACCCCGCTGCGTCGG<br>CTGATGGAGGCGTTTG<br>CAAAGCGCCAGGGTA<br>AAGAAATGGATTCTCT<br>GCGTTTCCTGTATGAT<br>GGCATCCGTATTCAAG<br>CAGACCAGACGCCAG<br>AGGATCTGGATATGGA<br>AGACAACGATATTATT<br>GAAGCGCATCGAGAG<br>CAAATCGGTGGTACCG<br>ATAGTAAGGACATGCA<br>GAGATTACAGACCACC<br>CAGCAGCGTGGTTAC<br>CCGTTGAATAGAGAAA<br>TGGAATTCCAGAAAAC<br>TACGGAGGTGCACAG<br>CATCAGCTCCGCGAG<br>CGAAGACGGTAGGAA<br>CGAGGTCCAGAGGTA<br>CACGGGCAAGATGTT<br>GGAGATGATTGTGGAA<br>CGTGGCAACATGGAA<br>GCTGCCTATAAGCGC<br>GTGGTTGCAAACAAGG<br>GCTCGCACGGTGTGG<br>ATGGTATGGGCGTTGA<br>CGAGTTGCTGCCGTAT<br>TTGAAGGAGAACTGG	HMSHHHHHHHHGGG<br>SGSSGGGSDSEVNQE<br>AKPEVKPEVKPETHINL<br>KVSDGSSEIFFKIKKTT<br>PLRRLMEAFAKRQGKE<br>MDSLRFLYDGIRIQAD<br>QTPEDLDMEDNDIIEA<br>HREQIGGTDSKDMQR<br>LQTTQQRGYPLNREM<br>EFQKTTEVHSISSASE<br>DGRNEVQRYTGKMLE<br>MIVERGNMEAAYKRVV<br>ANKGSHGVDGMGVDE<br>LLPYLKENWATIKQQLL<br>EGKYKPQPVRRVEIPK<br>PDGGKRLLGIPTVLDR<br>LIQQAIAQILNKVYNHT<br>FSDSSYGFRPGRSAK<br>DAIKAAEAYINEGYTW<br>VVDMDLEKFFDRVNH<br>DIIMSKLEKRIGDKRVL<br>KLIRRYLESGVMINGIK<br>VSTEEGTPQGGPLSPL<br>LANIMLDELDKELEKR<br>GHKFCRYADDCNIYVR<br>SRSAGNRVMKSIKKFIE<br>SKLKLKVNEAKSAVDR<br>PWRRKFLGFSFYTKEN<br>EVRIRIHEKSIKRFKEK<br>VREITNRNKGISMENRI<br>KRLNQITTGWVNYFGL<br>ADAKSIMKTLDEWIRR<br>RLRAMIWKQWKKIKTK<br>HDNLVKLGVEEQKAW<br>EYANTRKGYWRISNSP<br>ILNKTLTNKYFESIGYK<br>SLSQRYLIVHNS

		GCAACCATTAAACAAC<br>AACTGTTAGAGGGTAA<br>ATACAAGCCGCAACCG<br>GTGCGTAGAGTTGAGA<br>TCCCGAAACCGGATG<br>GTGGCAAACGCCTTCT<br>GGGAATCCCGACCGT<br>TCTGGACCGCTTGATC<br>CAACAAGCTATCGCCC<br>AGATTCTGAATAAAGT<br>GTATAACCACACCTTT<br>TCGGACTCCAGCTATG<br>GCTTTCGTCCAGGTCG<br>TTCTGCGAAAGATGCT<br>ATTAAGGCTGCGGAAG<br>CGTACATTAACGAGGG<br>TTATACCTGGGTTGTT<br>GATATGGACCTGGAGA<br>AGTTCTTCGATCGTGT<br>CAATCACGACATTATC<br>ATGAGCAAACTGGAGA<br>AACGCATCGGTGATAA<br>ACGTGTACTCAAGTTG<br>ATCCGTCGTTACTTGG<br>AGAGCGGCGTGATGA<br>TTAACGGTATCAAGGT<br>CAGCACCGAAGAGGG<br>CACTCCGCAGGGCGG<br>CCCCCTGAGCCCGCT<br>GTTGGCGAACATTATG<br>CTGGACGAGCTGGAC<br>AAAGAGCTGGAGAAG<br>CGTGGTCATAAATTCT<br>GTCGTTACGCTGACGA<br>CTGTAATATTTATGTTC<br>GTAGCCGTTCTGCGG<br>GTAACCGTGTTATGAA<br>AAGCATCAAAAAATTC<br>ATCGAGAGCAAACTGA<br>AGCTGAAAGTTAATGA<br>AGCGAAGTCGGCGGT<br>GGACCGTCCGTGGCG<br>CCGTAAGTTCCTTGGT<br>TTTAGCTTTTATACCAA<br>AGAGAACGAAGTTCGT<br>ATCCGTATTCACGAAA<br>AGAGCATCAAACGCTT<br>TAAGGAAAAGGTTCGC<br>GAAATCACCAATCGTA<br>ATAAGGGGATCAGCAT	

		GGAAAACCGTATCAAG<br>CGCCTCAACCAGATCA<br>CCACCGGCTGGGTCA<br>ACTATTTTGGTCTGGC<br>GGACGCGAAATCTATT<br>ATGAAAACGCTAGACG<br>AATGGATTCGCCGTCG<br>CTTACGTGCAATGATT<br>TGGAAACAATGGAAAA<br>AGATTAAGACCAAACA<br>TGATAATCTGGTTAAA<br>CTGGGTGTGGAGGAA<br>CAGAAAGCATGGGAAT<br>ACGCCAATACGCGCAA<br>AGGTTACTGGCGTATC<br>AGCAATAGCCCGATTC<br>TGAACAAGACCTTGAC<br>CAACAAGTACTTCGAG<br>TCGATCGGCTACAAAA<br>GCTTGTCTCAGCGCTA<br>CCTGATCGTCCACAAC<br>AGCTAATAGGATCC	
His-SUMO-TaitRT C2M (all)	Substitution of all cysteines to methionines in *Ta.it.*I1 IEP	CATATGTCACATCACC<br>ACCATCACCACCACCA<br>TGGAGGCGGTTCAGG<br>CTCTTCCGGTGGTGG<br>CTCCGACAGCGAAGT<br>GAACCAAGAAGCCAA<br>GCCGGAAGTTAAGCC<br>GGAAGTGAAGCCGGA<br>GACACATATCAACCTG<br>AAGGTGTCCGATGGTT<br>CCAGCGAGATTTTTTT<br>CAAGATCAAGAAAACG<br>ACCCCGCTGCGTCGG<br>CTGATGGAGGCGTTTG<br>CAAAGCGCCAGGGTA<br>AAGAAATGGATTCTCT<br>GCGTTTCCTGTATGAT<br>GGCATCCGTATTCAAG<br>CAGACCAGACGCCAG<br>AGGATCTGGATATGGA<br>AGACAACGATATTATT<br>GAAGCGCATCGAGAG<br>CAAATCGGTGGTACCG<br>ATAGTAAGGACATGCA<br>GAGATTACAGACCACC<br>CAGCAGCGTGGTTAC<br>CCGTTGAATAGAGAAA	HMSHHHHHHHHGGG<br>SGSSGGGSDSEVNQE<br>AKPEVKPEVKPETHINL<br>KVSDGSSEIFFKIKKTT<br>PLRRLMEAFAKRQGKE<br>MDSLRFLYDGIRIQAD<br>QTPEDLDMEDNDIIEA<br>HREQIGGTDSKDMQR<br>LQTTQQRGYPLNREM<br>EFQKTTEVHSISSASE<br>DGRNEVQRYTGKMLE<br>MIVERGNMEAAYKRVV<br>ANKGSHGVDGMGVDE<br>LLPYLKENWATIKQQLL<br>EGKYKPQPVRRVEIPK<br>PDGGKRLLGIPTVLDR<br>LIQQAIAQILNKVYNHT<br>FSDSSYGFRPGRSAK<br>DAIKAAEAYINEGYTW<br>VVDMDLEKFFDRVNH<br>DIIMSKLEKRIGDKRVL<br>KLIRRYLESGVMINGIK<br>VSTEEGTPQGGPLSPL<br>LANIMLDELDKELEKR<br>GHKFMRYADDMNIYV<br>RSRSAGNRVMKSIKKF<br>IESKLKLKVNEAKSAVD

		TGGAATTCCAGAAAAC TACGGAGGTGCACAG CATCAGCTCCGCGAG CGAAGACGGTAGGAA CGAGGTCCAGAGGTA CACGGGCAAGATGTT GGAGATGATTGTGGAA CGTGGCAACATGGAA GCTGCCTATAAGCGC GTGGTTGCAAACAAGG GCTCGCACGGTGTGG ATGGTATGGGCGTTGA CGAGTTGCTGCCGTAT TTGAAGGAGAACTGG GCAACCATTAAACAAC AACTGTTAGAGGGTAA ATACAAGCCGCAACCG GTGCGTAGAGTTGAGA TCCCGAAACCGGATG GTGGCAAACGCCTTCT GGGAATCCCGACCGT TCTGGACCGCTTGATC CAACAAGCTATCGCCC AGATTCTGAATAAAGT GTATAACCACACCTTT TCGGACTCCAGCTATG GCTTTCGTCCAGGTCG TTCTGCGAAAGATGCT ATTAAGGCTGCGGAAG CGTACATTAACGAGGG TTATACCTGGGTTGTT GATATGGACCTGGAGA AGTTCTTCGATCGTGT CAATCACGACATTATC ATGAGCAAACTGGAGA AACGCATCGGTGATAA ACGTGTACTCAAGTTG ATCCGTCGTTACTTGG AGAGCGGCGTGATGA TTAACGGTATCAAGGT CAGCACCGAAGAGGG CACTCCGCAGGGCGG CCCCCTGAGCCCGCT GTTGGCGAACATTATG CTGGACGAGCTGGAC AAAGAGCTGGAGAAG CGTGGTCATAAATTCA TGCGTTACGCTGACGA CATGAATATTTATGTTC GTAGCCGTTCTGCGG	RPWRRKFLGFSFYTKE NEVRIRIHEKSIKRFKE KVREITNRNKGISMEN RIKRLNQITTGWVNYF GLADAKSIMKTLDEWI RRRLRAMIWKQWKKIK TKHDNLVKLGVEEQKA WEYANTRKGYWRISN SPILNKTLTNKYFESIG YKSLSQRYLIVHNS

		GTAACCGTGTTATGAA AAGCATCAAAAAATTC ATCGAGAGCAAACTGA AGCTGAAAGTTAATGA AGCGAAGTCGGCGGT GGACCGTCCGTGGCG CCGTAAGTTCCTTGGT TTTAGCTTTTATACCAA AGAGAACGAAGTTCGT ATCCGTATTCACGAAA AGAGCATCAAACGCTT TAAGGAAAAGGTTCGC GAAATCACCAATCGTA ATAAGGGGATCAGCAT GGAAAACCGTATCAAG CGCCTCAACCAGATCA CCACCGGCTGGGTCA ACTATTTTGGTCTGGC GGACGCGAAATCTATT ATGAAAACGCTAGACG AATGGATTCGCCGTCG CTTACGTGCAATGATT TGGAAACAATGGAAAA AGATTAAGACCAAACA TGATAATCTGGTTAAA CTGGGTGTGGAGGAA CAGAAAGCATGGGAAT ACGCCAATACGCGCAA AGGTTACTGGCGTATC AGCAATAGCCCGATTC TGAACAAGACCTTGAC CAACAAGTACTTCGAG TCGATCGGCTACAAAA GCTTGTCTCAGCGCTA CCTGATCGTCCACAAC AGCTAATAGGATCC	
His-SUMO-TaitRT C2S (all)	Substitution of all cysteines to serines in *Ta.it.*I1 IEP	CATATGTCACATCACC ACCATCACCACCACCA TGGAGGCGGTTCAGG CTCTTCCGGTGGTGG CTCCGACAGCGAAGT GAACCAAGAAGCCAA GCCGGAAGTTAAGCC GGAAGTGAAGCCGGA GACACATATCAACCTG AAGGTGTCCGATGGTT CCAGCGAGATTTTTTT CAAGATCAAGAAAACG ACCCCGCTGCGTCGG	HMSHHHHHHHHGGG SGSSGGGSDSEVNQE AKPEVKPEVKPETHINL KVSDGSSEIFFKIKKTT PLRRLMEAFAKRQGKE MDSLRFLYDGIRIQAD QTPEDLDMEDNDIIEA HREQIGGTDSKDMQR LQTTQQRGYPLNREM EFQKTTEVHSISSASE DGRNEVQRYTGKMLE MIVERGNMEAAYKRVV ANKGSHGVDGMGVDE

		CTGATGGAGGCGTTTG<br>CAAAGCGCCAGGGTA<br>AAGAAATGGATTCTCT<br>GCGTTTCCTGTATGAT<br>GGCATCCGTATTCAAG<br>CAGACCAGACGCCAG<br>AGGATCTGGATATGGA<br>AGACAACGATATTATT<br>GAAGCGCATCGAGAG<br>CAAATCGGTGGTACCG<br>ATAGTAAGGACATGCA<br>GAGATTACAGACCACC<br>CAGCAGCGTGGTTAC<br>CCGTTGAATAGAGAAA<br>TGGAATTCCAGAAAAC<br>TACGGAGGTGCACAG<br>CATCAGCTCCGCGAG<br>CGAAGACGGTAGGAA<br>CGAGGTCCAGAGGTA<br>CACGGGCAAGATGTT<br>GGAGATGATTGTGGAA<br>CGTGGCAACATGGAA<br>GCTGCCTATAAGCGC<br>GTGGTTGCAAACAAGG<br>GCTCGCACGGTGTGG<br>ATGGTATGGGCGTTGA<br>CGAGTTGCTGCCGTAT<br>TTGAAGGAGAACTGG<br>GCAACCATTAAACAAC<br>AACTGTTAGAGGGTAA<br>ATACAAGCCGCAACCG<br>GTGCGTAGAGTTGAGA<br>TCCCGAAACCGGATG<br>GTGGCAAACGCCTTCT<br>GGGAATCCCGACCGT<br>TCTGGACCGCTTGATC<br>CAACAAGCTATCGCCC<br>AGATTCTGAATAAAGT<br>GTATAACCACACCTTT<br>TCGGACTCCAGCTATG<br>GCTTTCGTCCAGGTCG<br>TTCTGCGAAAGATGCT<br>ATTAAGGCTGCGGAAG<br>CGTACATTAACGAGGG<br>TTATACCTGGGTTGTT<br>GATATGGACCTGGAGA<br>AGTTCTTCGATCGTGT<br>CAATCACGACATTATC<br>ATGAGCAAACTGGAGA<br>AACGCATCGGTGATAA	LLPYLKENWATIKQQLL<br>EGKYKPQPVRRVEIPK<br>PDGGKRLLGIPTVLDR<br>LIQQAIAQILNKVYNHT<br>FSDSSYGFRPGRSAK<br>DAIKAAEAYINEGYTW<br>VVDMDLEKFFDRVNH<br>DIIMSKLEKRIGDKRVL<br>KLIRRYLESGVMINGIK<br>VSTEEGTPQGGPLSPL<br>LANIMLDELDKELEKR<br>GHKFSRYADDSNIYVR<br>SRSAGNRVMKSIKKFIE<br>SKLKLKVNEAKSAVDR<br>PWRRKFLGFSFYTKEN<br>EVRIRIHEKSIKRFKEK<br>VREITNRNKGISMENRI<br>KRLNQITTGWVNYFGL<br>ADAKSIMKTLDEWIRR<br>RLRASIWKQWKKIKTK<br>HDNLVKLGVEEQKAW<br>EYANTRKGYWRISNSP<br>ILNKTLTNKYFESIGYK<br>SLSQRYLIVHNS

		ACGTGTACTCAAGTTG ATCCGTCGTTACTTGG AGAGCGGCGTGATGA TTAACGGTATCAAGGT CAGCACCGAAGAGGG CACTCCGCAGGGCGG CCCCCTGAGCCCGCT GTTGGCGAACATTATG CTGGACGAGCTGGAC AAAGAGCTGGAGAAG CGTGGTCATAAATTCT CTCGTTACGCTGACGA CTCTAATATTTATGTTC GTAGCCGTTCTGCGG GTAACCGTGTTATGAA AAGCATCAAAAAATTC ATCGAGAGCAAACTGA AGCTGAAAGTTAATGA AGCGAAGTCGGCGGT GGACCGTCCGTGGCG CCGTAAGTTCCTTGGT TTTAGCTTTTATACCAA AGAGAACGAAGTTCGT ATCCGTATTCACGAAA AGAGCATCAAACGCTT TAAGGAAAAGGTTCGC GAAATCACCAATCGTA ATAAGGGGATCAGCAT GGAAAACCGTATCAAG CGCCTCAACCAGATCA CCACCGGCTGGGTCA ACTATTTTGGTCTGGC GGACGCGAAATCTATT ATGAAAACGCTAGACG AATGGATTCGCCGTCG CTTACGTGCATCTATT TGGAAACAATGGAAAA AGATTAAGACCAAACA TGATAATCTGGTTAAA CTGGGTGTGGAGGAA CAGAAAGCATGGGAAT ACGCCAATACGCGCAA AGGTTACTGGCGTATC AGCAATAGCCCGATTC TGAACAAGACCTTGAC CAACAAGTACTTCGAG TCGATCGGCTACAAAA GCTTGTCTCAGCGCTA CCTGATCGTCCACAAC AGCTAATAGGATCC	

His-SUMOTaitRT D4 contact	E271C<br>*Ta.it.*I1 IEP mutant	CATATGTCACATCACC<br>ACCATCACCACCACCA<br>TGGAGGCGGTTCAGG<br>CTCTTCCGGTGGTGG<br>CTCCGACAGCGAAGT<br>GAACCAAGAAGCCAA<br>GCCGGAAGTTAAGCC<br>GGAAGTGAAGCCGGA<br>GACACATATCAACCTG<br>AAGGTGTCCGATGGTT<br>CCAGCGAGATTTTTTT<br>CAAGATCAAGAAAACG<br>ACCCCGCTGCGTCGG<br>CTGATGGAGGCGTTTG<br>CAAAGCGCCAGGGTA<br>AAGAAATGGATTCTCT<br>GCGTTTCCTGTATGAT<br>GGCATCCGTATTCAAG<br>CAGACCAGACGCCAG<br>AGGATCTGGATATGGA<br>AGACAACGATATTATT<br>GAAGCGCATCGAGAG<br>CAAATCGGTGGTACCG<br>ATAGTAAGGACATGCA<br>GAGATTACAGACCACC<br>CAGCAGCGTGGTTAC<br>CCGTTGAATAGAGAAA<br>TGGAATTCCAGAAAAC<br>TACGGAGGTGCACAG<br>CATCAGCTCCGCGAG<br>CGAAGACGGTAGGAA<br>CGAGGTCCAGAGGTA<br>CACGGGCAAGATGTT<br>GGAGATGATTGTGGAA<br>CGTGGCAACATGGAA<br>GCTGCCTATAAGCGC<br>GTGGTTGCAAACAAGG<br>GCTCGCACGGTGTGG<br>ATGGTATGGGCGTTGA<br>CGAGTTGCTGCCGTAT<br>TTGAAGGAGAACTGG<br>GCAACCATTAAACAAC<br>AACTGTTAGAGGGTAA<br>ATACAAGCCGCAACCG<br>GTGCGTAGAGTTGAGA<br>TCCCGAAACCGGATG<br>GTGGCAAACGCCTTCT<br>GGGAATCCCGACCGT<br>TCTGGACCGCTTGATC<br>CAACAAGCTATCGCCC	HMSHHHHHHHHGGG<br>SGSSGGGSDSEVNQE<br>AKPEVKPEVKPETHINL<br>KVSDGSSEIFFKIKKTT<br>PLRRLMEAFAKRQGKE<br>MDSLRFLYDGIRIQAD<br>QTPEDLDMEDNDIIEA<br>HREQIGGTDSKDMQR<br>LQTTQQRGYPLNREM<br>EFQKTTEVHSISSASE<br>DGRNEVQRYTGKMLE<br>MIVERGNMEAAYKRVV<br>ANKGSHGVDGMGVDE<br>LLPYLKENWATIKQQLL<br>EGKYKPQPVRRVEIPK<br>PDGGKRLLGIPTVLDR<br>LIQQAIAQILNKVYNHT<br>FSDSSYGFRPGRSAK<br>DAIKAAEAYINEGYTW<br>VVDMDLEKFFDRVNH<br>DIIMSKLEKRIGDKRVL<br>KLIRRYLESGVMINGIK<br>VSTEEGTPQGGPLSPL<br>LANIMLDELDKELEKR<br>GHKFMRYADDMNIYV<br>RSRSAGNRVMKSIKKF<br>IESKLKLKVNEAKSAVD<br>RPWRRKFLGFSFYTKC<br>NEVRIRIHEKSIKRFKE<br>KVREITNRNKGISMEN<br>RIKRLNQITTGWVNYF<br>GLADAKSIMKTLDEWI<br>RRRLRAMIWKQWKKIK<br>TKHDNLVKLGVEEQKA<br>WEYANTRKGYWRISN<br>SPILNKTLTNKYFESIG<br>YKSLSQRYLIVHNS

		AGATTCTGAATAAAGT<br>GTATAACCACACCTTT<br>TCGGACTCCAGCTATG<br>GCTTTCGTCCAGGTCG<br>TTCTGCGAAAGATGCT<br>ATTAAGGCTGCGGAAG<br>CGTACATTAACGAGGG<br>TTATACCTGGGTTGTT<br>GATATGGACCTGGAGA<br>AGTTCTTCGATCGTGT<br>CAATCACGACATTATC<br>ATGAGCAAACTGGAGA<br>AACGCATCGGTGATAA<br>ACGTGTACTCAAGTTG<br>ATCCGTCGTTACTTGG<br>AGAGCGGCGTGATGA<br>TTAACGGTATCAAGGT<br>CAGCACCGAAGAGGG<br>CACTCCGCAGGGCGG<br>CCCCCTGAGCCCGCT<br>GTTGGCGAACATTATG<br>CTGGACGAGCTGGAC<br>AAAGAGCTGGAGAAG<br>CGTGGTCATAAATTCA<br>TGCGTTACGCTGACGA<br>CATGAATATTTATGTTC<br>GTAGCCGTTCTGCGG<br>GTAACCGTGTTATGAA<br>AAGCATCAAAAAATTC<br>ATCGAGAGCAAACTGA<br>AGCTGAAAGTTAATGA<br>AGCGAAGTCGGCGGT<br>GGACCGTCCGTGGCG<br>CCGTAAGTTCCTTGGT<br>TTTAGCTTTTATACCAA<br>ATGTAACGAAGTTCGT<br>ATCCGTATTCACGAAA<br>AGAGCATCAAACGCTT<br>TAAGGAAAAGGTTCGC<br>GAAATCACCAATCGTA<br>ATAAGGGGATCAGCAT<br>GGAAAACCGTATCAAG<br>CGCCTCAACCAGATCA<br>CCACCGGCTGGGTCA<br>ACTATTTTGGTCTGGC<br>GGACGCGAAATCTATT<br>ATGAAAACGCTAGACG<br>AATGGATTCGCCGTCG<br>CTTACGTGCAATGATT<br>TGGAAACAATGGAAAA	

		AGATTAAGACCAAACA<br>TGATAATCTGGTTAAA<br>CTGGGTGTGGAGGAA<br>CAGAAAGCATGGGAAT<br>ACGCCAATACGCGCAA<br>AGGTTACTGGCGTATC<br>AGCAATAGCCCGATTC<br>TGAACAAGACCTTGAC<br>CAACAAGTACTTCGAG<br>TCGATCGGCTACAAAA<br>GCTTGTCTCAGCGCTA<br>CCTGATCGTCCACAAC<br>AGCTAATAGGATCC	
His-SUMO-TaitRT YAAA	Mutation of catalytic motif YADD to YAAA in *Ta.it.*I1 IEP	CATATGTCAGGCCATC<br>ACCATCACCATCACCA<br>TCATGGTGGAGGGAG<br>TGGCTCGTCTGGCGG<br>GGGTTCTGGAATGTCA<br>GACTCGGAGGTGAAC<br>CAGGAAGCGAAGCCG<br>GAAGTTAAGCCG<br>GAAGTCAAGCCTGAGA<br>CGCACATTAATCTGAA<br>AGTAAGTGATGGTTCC<br>TCTGAGATCTTT<br>TTTAAGATTAAAAAAC<br>CACCCCTCTTCGTCGT<br>TTGATGGAGGCATTTG<br>CCAAACGTCAA<br>GGGAAGGAAATGGAC<br>TCATTGCGTTTCCTTTA<br>TGATGGTATCCGCATT<br>CAGGCAGATCAA<br>ACCCCGGAAGATTTGG<br>ACATGGAGGACAATGA<br>TATTATCGAGGCGCAT<br>CGCGAACAAATC<br>GGTGGCGCGGCCGCG<br>GAAATGGACAGCAAG<br>GATATGCAACGTTTAC<br>AAACTACTCAACAG<br>CGCGGTTACCCTCTGA<br>ATCGCGAGATGGAGTT<br>CCAAAAGACCACTGAG<br>GTACACTCTATT<br>TCCTCCGCTTCCGAAG<br>ATGGGCGTAACGAAGT<br>ACAGCGCTACACGGG<br>TAAGATGCTTGAG	HMSGHHHHHHHHGG<br>GSGSSGGGSGMSDSE<br>VNQEAKPEVKPEVKPE<br>THINLKVSDGSSEIFFKI<br>KKTTPLRRLMEAFAKR<br>QGKEMDSLRFLYDGIR<br>IQADQTPEDLDMEDND<br>IIEAHREQIGGAAAEMD<br>SKDMQRLQTTQQRGY<br>PLNREMEFQKTTEVHS<br>ISSASEDGRNEVQRYT<br>GKMLEMIVERGNMEA<br>AYKRVVANKGSHGVD<br>GMGVDELLPYLKENW<br>ATIKQQLLEGKYKPQP<br>VRRVEIPKPDGGKRLL<br>GIPTVLDRLIQQAIAQIL<br>NKVYNHTFSDSSYGFR<br>PGRSAKDAIKAAEAYIN<br>EGYTWVVDMDLEKFF<br>DRVNHDIIMSKLEKRIG<br>DKRVLKLIRRYLESGV<br>MINGIKVSTEEGTPQG<br>GPLSPLLANIMLDELDK<br>ELEKRGHKFCRYAAAC<br>NIYVRSRSAGNRVMKS<br>IKKFIESKLKLKVNEAK<br>SAVDRPWRRKFLGFS<br>FYTKENEVRIRIHEKSIK<br>RFKEKVREITNRNKGIS<br>MENRIKRLNQITTGWV<br>NYFGLADAKSIMKTLD<br>EWIRRRLRACIWKQW<br>KKIKTKHDNLVKLGVEE<br>QKAWEYANTRKGYWR<br>ISNSPILNKTLTNKYFE

		ATGATCGTCGAACGTG<br>GAAATATGGAGGCAG<br>CTTACAAGCGTGTTGT<br>TGCAAATAAAGGG<br>TCCCACGGGGTAGAC<br>GGTATGGGCGTCGAT<br>GAATTGCTGCCGTACT<br>TAAAAGAAAATTGG<br>GCCACAATCAAACAAC<br>AGTTGTTAGAAGGCAA<br>GTACAAACCCCAACCC<br>GTCCGTCGTGTT<br>GAAATCCCTAAGCCAG<br>ATGGAGGAAAACGTCT<br>TCTGGGGATTCCAACC<br>GTATTAGATCGT<br>CTTATTCAACAGGCAA<br>TTGCACAGATTTTGAA<br>TAAGGTATATAATCATA<br>CTTTTTCCGAC<br>AGTTCCTATGGTTTTC<br>GTCCCGGGCGCTCTG<br>CGAAAGACGCTATTAA<br>GGCTGCTGAGGCC<br>TATATTAACGAGGGCT<br>ATACCTGGGTCGTGGA<br>TATGGACTTGGAGAAG<br>TTCTTTGATCGC<br>GTGAATCACGACATTA<br>TTATGTCCAAGCTTGA<br>AAAGCGCATCGGCGA<br>TAAGCGTGTTCTT<br>AAATTGATTCGCCGCT<br>ATTTGGAGTCCGGAGT<br>AATGATCAATGGCATC<br>AAGGTTAGCACT<br>GAAGAAGGTACACCAC<br>AAGGAGGCCCACTTA<br>GCCCTCTGTTAGCTAA<br>TATTATGTTGGAT<br>GAGCTGGACAAAGAG<br>CTTGAGAAACGCGGTC<br>ACAAGTTCTGTCGTTA<br>CGCGGCGGCCTGC<br>AATATCTATGTGCGCA<br>GTCGCTCGGCTGGTA<br>ACCGCGTCATGAAAAG<br>CATTAAGAAGTTC<br>ATCGAAAGTAAATTAA<br>AGCTTAAGGTAAATGA	SIGYKSLSQRYLIVHNS

		AGCGAAATCGGCGGT GGACCGTCCGTGG CGTCGTAAGTTCCTGG GTTTCAGTTTTTATACG AAAGAGAATGAAGTAC GCATTCGTATC CACGAGAAATCCATCA AACGTTTTAAGGAAAA AGTCCGTGAGATTACG AACCGTAACAAG GGAATTAGCATGGAAA ACCGCATTAAGCGCCT TAATCAAATTACTACTG GCTGGGTAAAT TACTTTGGATTAGCGG ACGCCAAGTCAATTAT GAAAACACTGGATGAA TGGATTCGTCGT CGTTTGCGTGCCTGCA TTTGGAAGCAATGGAA AAAGATCAAAACGAAA CACGACAACTTG GTAAAGCTTGGAGTGG AGGAACAAAAGGCGT GGGAATATGCCAACAC ACGTAAAGGTTAC TGGCGTATCTCCAACT CACCAATTCTTAATAA GACCCTTACAAATAAA TACTTCGAATCA ATTGGGTACAAGTCAC TGTCCCAACGCTATTT AATTGTTCATAATTCGT AATAGGGATCC	
His-SUMO-TelRT	Wild-type *T.el* IEP	CATATGGGTAGCCATC ATCACCACCACCACCA CCATGGCGGTGGCAG CGGCAGCAGCGGTGG CGGTAGCGATAGCGA AGTGAACCAAGAGGC GAAGCCGGAAGTGAA ACCGGAAGTGAAGCC GGAAACCCACATTAAC CTGAAAGTTAGCGACG GTAGCAGCGAAATCTT CTTTAAGATTAAGAAA ACCACCCCGCTGCGT CGTCTGATGGAGGCG TTCGCGAAACGTCAGG	HMGSHHHHHHHHGG GSGSSGGGSDSEVNQ EAKPEVKPEVKPETHI NLKVSDGSSEIFFKIKK TTPLRRLMEAFAKRQG KEMDSLRFLYDGIRIQA DQTPEDLDMEDNDIIE AHREQIGGTETRQMAV EQTTGAVTNQTETSW HSIDWAKANREVKRLQ VRIAKAVKEGRWGKVK ALQWLLTHSFYGKALA VKRVTDNSGSKTPGV DGITWSTQEQKAQAIK SLRRRGYKPQPLRRVY

		GCAAGGAAATGGACA<br>GCCTGCGTTTTCTGTA<br>TGATGGTATCCGTATT<br>CAGGCGGACCAAACC<br>CCGGAGGACCTGGAT<br>ATGGAAGACAACGATA<br>TCATTGAGGCGCACC<br>GTGAACAGATCGGTG<br>GTACCGAGACCCGTC<br>AGATGGCGGTGGAAC<br>AAACCACCGGTGCGG<br>TTACCAACCAAACCGA<br>GACCAGCTGGCACAG<br>CATTGATTGGGCGAAG<br>GCGAACCGTGAAGTG<br>AAACGTCTGCAGGTTC<br>GTATCGCGAAGGCGG<br>TGAAAGAGGGCCGTT<br>GGGGCAAGGTTAAAG<br>CGCTGCAATGGCTGCT<br>GACCCACAGCTTCTAC<br>GGCAAGGCGCTGGCG<br>GTGAAACGTGTTACCG<br>ATAACAGCGGTAGCAA<br>AACCCCGGGCGTGGA<br>CGGTATTACCTGGAGC<br>ACCCAGGAGCAAAAG<br>GCGCAGGCGATCAAA<br>AGCCTGCGTCGTCGT<br>GGTTACAAACCGCAAC<br>CGCTGCGTCGTGTTTA<br>TATTCCGAAAGCGAGC<br>GGCAAGCAGCGTCCG<br>CTGGGTATCCCGACCA<br>CCAAGGATCGTGCGAT<br>GCAAGCGCTGTATGC<br>GCTGGCGCTGGAGCC<br>GGTGGCGGAAACCAC<br>CGCGGACCGTAACAG<br>CTATGGCTTCCGTCAA<br>GGTCGTTGCACCGCG<br>GATGCGGCGGGTCAA<br>TGCTTTACCGTTCTGG<br>GTCGTAGCGACTGCG<br>CGAAGTACATCCTGGA<br>CGCGGATATTACCGGT<br>TGCTTTGATAACATTA<br>GCCACGAGTGGCTGC<br>TGGACAACATCCCGCT<br>GGATAAAGAAGTGCTG	IPKASGKQRPLGIPTTK<br>DRAMQALYALALEPVA<br>ETTADRNSYGFRQGR<br>CTADAAGQCFTVLGRS<br>DCAKYILDADITGCFDN<br>ISHEWLLDNIPLDKEVL<br>RKWLKSGFVWKQQLF<br>PTHAGTPQGGVISPML<br>ANMTLDGMEELLKKHL<br>RKQKVNLIRYADDFVV<br>TGESKETLEKVTTVIQE<br>FLKERGLTLSEEKTKV<br>VHIEEGFDFLGWNIRK<br>YGEKLLIKPAKKNIKAF<br>HKKIRDALKELRTATQE<br>AVIDTLNPIIKGWANYH<br>RNQVSKRIFNRADDNI<br>WHKLWRWAKRRHPN<br>KPARWTKNKYFIKIGN<br>RHWVFGTWKKDKEGR<br>LRSRYLIKAGDTRIQRH<br>VKIKADANPFLPEWAE<br>YFEERKKLKEAPAQYR<br>RIRRELWKKQGGICPV<br>CGGEIEQDMLTEIHHIL<br>PKHKGGSDDLDNLVLI<br>HANCHKQVHSRDGQH<br>SRFLLKEGL

		CGTAAGTGGCTGAAAA GCGGCTTCGTTTGGAA GCAGCAACTGTTTCCG ACCCATGCGGGCACC CCGCAGGGTGGTGTG ATCAGCCCGATGCTG GCGAACATGACCCTG GACGGTATGGAGGAA CTGCTGAAGAAACACC TGCGTAAGCAAAAAGT TAACCTGATTCGTTAT GCGGACGATTTCGTG GTTACCGGCGAGAGC AAGGAAACCCTGGAG AAAGTGACCACCGTTA TCCAGGAGTTTCTGAA AGAACGTGGTCTGACC CTGAGCGAGGAAAAG ACCAAAGTGGTTCACA TTGAGGAAGGCTTCGA CTTTCTGGGTTGGAAC ATCCGTAAATACGGCG AAAAGCTGCTGATCAA ACCGGCGAAGAAAAA CATTAAAGCGTTCCAC AAGAAAATCCGTGATG CGCTGAAGGAGCTGC GTACCGCGACCCAGG AAGCGGTGATCGACA CCCTGAACCCGATCAT TAAAGGTTGGGCGAAC TATCACCGTAACCAAG TTAGCAAGCGTATTTT TAACCGTGCGGACGAT AACATCTGGCACAAGC TGTGGCGTTGGGCGA AACGTCGTCACCCGAA CAAGCCGGCGCGTTG GACCAAGAACAAATAC TTCATCAAAATTGGCA ACCGTCACTGGGTGTT TGGCACCTGGAAGAAA GATAAAGAGGGCCGT CTGCGTAGCCGTTATC TGATTAAGGCGGGTGA TACCCGTATCCAGCGT CACGTTAAGATTAAAG CGGACGCGAACCCGT TCCTGCCGGAATGGG CGGAGTACTTTGAGGA	

		GCGTAAGAAACTGAAA GAGGCGCCGGCGCAG TATCGTCGTATCCGTC GTGAACTGTGGAAGAA ACAAGGCGGTATTTGC CCGGTTTGCGGCGGT GAGATCGAACAGGAC ATGCTGACCGAAATCC ACCACATTCTGCCGAA GCACAAAGGCGGTAG CGACGATCTGGATAAC CTGGTGCTGATCCACG CGAACTGCCACAAACA GGTTCACAGCCGTGA CGGCCAACACAGCCG TTTCCTGCTGAAAGAG GGTCTGTAAGGATCC	
His-SUMO-TelRT C2M (all)	Substitution of all cysteines to methionines in *T.el* IEP	CATATGGGTAGCCATC ATCACCACCACCACCA CCATGGCGGTGGCAG CGGCAGCAGCGGTGG CGGTAGCGATAGCGA AGTGAACCAAGAGGC GAAGCCGGAAGTGAA ACCGGAAGTGAAGCC GGAAACCCACATTAAC CTGAAAGTTAGCGACG GTAGCAGCGAAATCTT CTTTAAGATTAAGAAA ACCACCCCGCTGCGT CGTCTGATGGAGGCG TTCGCGAAACGTCAGG GCAAGGAAATGGACA GCCTGCGTTTTCTGTA TGATGGTATCCGTATT CAGGCGGACCAAACC CCGGAGGACCTGGAT ATGGAAGACAACGATA TCATTGAGGCGCACC GTGAACAGATCGGTG GTACCGAGACCCGTC AGATGGCGGTGGAAC AAACCACCGGTGCGG TTACCAACCAAACCGA GACCAGCTGGCACAG CATTGATTGGGCGAAG GCGAACCGTGAAGTG AAACGTCTGCAGGTTC GTATCGCGAAGGCGG	HMGSHHHHHHHHGG GSGSSGGGSDSEVNQ EAKPEVKPEVKPETHI NLKVSDGSSEIFFKIKK TTPLRRLMEAFAKRQG KEMDSLRFLYDGIRIQA DQTPEDLDMEDNDIIE AHREQIGGTETRQMAV EQTTGAVTNQTETSW HSIDWAKANREVKRLQ VRIAKAVKEGRWGKVK ALQWLLTHSFYGKALA VKRVTDNSGSKTPGV DGITWSTQEQKAQAIK SLRRRGYKPQPLRRVY IPKASGKQRPLGIPTTK DRAMQALYALALEPVA ETTADRNSYGFRQGR MTADAAGQSFTVLGR SDMAKYILDADITGMF DNISHEWLLDNIPLDKE VLRKWLKSGFVWKQQ LFPTHAGTPQGGVISP MLANMTLDGMEELLKK HLRKQKVNLIRYADDF VVTGESKETLEKVTTVI QEFLKERGLTLSEEKT KVVHIEEGFDFLGWNI RKYGEKLLIKPAKKNIK AFHKKIRDALKELRTAT QEAVIDTLNPIIKGWAN YHRNQVSKRIFNRADD

		TGAAAGAGGGCCGTT GGGGCAAGGTTAAAG CGCTGCAATGGCTGCT GACCCACAGCTTCTAC GGCAAGGCGCTGGCG GTGAAACGTGTTACCG ATAACAGCGGTAGCAA AACCCCGGGCGTGGA CGGTATTACCTGGAGC ACCCAGGAGCAAAAG GCGCAGGCGATCAAA AGCCTGCGTCGTCGT GGTTACAAACCGCAAC CGCTGCGTCGTGTTTA TATTCCGAAAGCGAGC GGCAAGCAGCGTCCG CTGGGTATCCCGACCA CCAAGGATCGTGCGAT GCAAGCGCTGTATGC GCTGGCGCTGGAGCC GGTGGCGGAAACCAC CGCGGACCGTAACAG CTATGGCTTCCGTCAA GGTCGTATGACCGCG GATGCGGCGGGTCAA AGTTTTACCGTTCTGG GTCGTAGCGACATGG CGAAGTACATCCTGGA CGCGGATATTACCGGT ATGTTTGATAACATTA GCCACGAGTGGCTGC TGGACAACATCCCGCT GGATAAAGAAGTGCTG CGTAAGTGGCTGAAAA GCGGCTTCGTTTGGAA GCAGCAACTGTTTCCG ACCCATGCGGGCACC CCGCAGGGTGGTGTG ATCAGCCCGATGCTG GCGAACATGACCCTG GACGGTATGGAGGAA CTGCTGAAGAAACACC TGCGTAAGCAAAAAGT TAACCTGATTCGTTAT GCGGACGATTTCGTG GTTACCGGCGAGAGC AAGGAAACCCTGGAG AAAGTGACCACCGTTA TCCAGGAGTTTCTGAA AGAACGTGGTCTGACC	NIWHKLWRWAKRRHP NKPARWTKNKYFIKIG NRHWVFGTWKKDKEG RLRSRYLIKAGDTRIQR HVKIKADANPFLPEWA EYFEERKKLKEAPAQY RRIRRELWKKQGGIMP VMGGEIEQDMLTEIHHI LPKHKGGSDDLDNLVL IHANMHKQVHSRDGQ HSRFLLKEGL

		CTGAGCGAGGAAAAG<br>ACCAAAGTGGTTCACA<br>TTGAGGAAGGCTTCGA<br>CTTTCTGGGTTGGAAC<br>ATCCGTAAATACGGCG<br>AAAAGCTGCTGATCAA<br>ACCGGCGAAGAAAAA<br>CATTAAAGCGTTCCAC<br>AAGAAAATCCGTGATG<br>CGCTGAAGGAGCTGC<br>GTACCGCGACCCAGG<br>AAGCGGTGATCGACA<br>CCCTGAACCCGATCAT<br>TAAAGGTTGGGCGAAC<br>TATCACCGTAACCAAG<br>TTAGCAAGCGTATTTT<br>TAACCGTGCGGACGAT<br>AACATCTGGCACAAGC<br>TGTGGCGTTGGGCGA<br>AACGTCGTCACCCGAA<br>CAAGCCGGCGCGTTG<br>GACCAAGAACAAATAC<br>TTCATCAAAATTGGCA<br>ACCGTCACTGGGTGTT<br>TGGCACCTGGAAGAAA<br>GATAAAGAGGGCCGT<br>CTGCGTAGCCGTTATC<br>TGATTAAGGCGGGTGA<br>TACCCGTATCCAGCGT<br>CACGTTAAGATTAAAG<br>CGGACGCGAACCCGT<br>TCCTGCCGGAATGGG<br>CGGAGTACTTTGAGGA<br>GCGTAAGAAACTGAAA<br>GAGGCGCCGGCGCAG<br>TATCGTCGTATCCGTC<br>GTGAACTGTGGAAGAA<br>ACAAGGCGGTATTATG<br>CCGGTTATGGGCGGT<br>GAGATCGAACAGGAC<br>ATGCTGACCGAAATCC<br>ACCACATTCTGCCGAA<br>GCACAAAGGCGGTAG<br>CGACGATCTGGATAAC<br>CTGGTGCTGATCCACG<br>CGAACATGCACAAACA<br>GGTTCACAGCCGTGA<br>CGGCCAACACAGCCG<br>TTTCCTGCTGAAAGAG<br>GGTCTGTAAGGATCC	

Supplementary Table 2: List of all RNA sequences used in this study

Construct Name	Description	Sequence
Tait WT RNA	Wild-type of *Ta.it.*I1 intron RNA	CUAAGACUACGAGCAUCU<br>GGCGUAAUACGACUCACU<br>AUAGGGCCGCAGUCUAAA<br>AGUAAUUUUAGACUGCUU<br>UUUUAUGUGCGCCCGGCA<br>UGGGCGAUAACUAGGCGG<br>UGAAAGUCCGCUGUGGGC<br>UUGGUAGUGGGAACCACU<br>AGCCAAGAGCAAGGGUGU<br>CCAUCGUGAGGUGGAAUC<br>UGAAGGAAGCUUAAGGCA<br>AAAUCUCGGUCUGAUGAA<br>CAAGAACCAGAUAAGAGG<br>CUGAAUUGGGAUGGAUGA<br>GUUUGCGUAACAAAACGA<br>AGUCCAACACUACCCGAA<br>UCCCAUACAGUAAAUCUG<br>GCAGAUAUAUGAGAUGAA<br>AGUUAUCGUUCUUACCCG<br>GGGAGGUCUCAAGGAUAA<br>GUCAUGGGAGUAAAAUCU<br>GAAGUGACAACCCAUGCA<br>GUGAUGUAUGGCUGAACC<br>UUGAGAAGUCAGCAGAGG<br>UCAUAGUACUUAUCUAGA<br>CAUGAAUAGAUAAGGAAG<br>GACCGAACGUUAGGAGGU<br>UUUGGAAAUCUUAUGGAC<br>UCGAAAGAUAUGCAGAGA<br>CUGCAGACAACUCAACAA<br>AGAGGCUAUCCGUUGUUC<br>GCAACGGAAGUGCAUAGU<br>AUAUCAUCGGCGUCGGAA<br>GAUGGAAGAAACUCCUAA<br>UGAACCGCCGUAUACCGA<br>ACGGUACGUACGGUGGU<br>GUGAGAGGACGCUGAAUA<br>AAAUAAUUAUUCAGCUCC<br>UACUCGAUUCAUGGAUCC<br>UGUUG
Tel WT RNA	Wild-type of *T.el* intron RNA	CUAAGACUACGAGCAUCU<br>GGCGUAAUACGACUCACU<br>AUAGGGAUAAUGAUUUUC

		CCAGGGUUGGCCGAGCG UUGCGACGCGAAAGCUAG CCAGAUGAUUGUCCCACU AGCCCAACAAGCUAGAAC GGGACCGGUUGUUCCCC CAACCGUAGCCUAGGGAG GCAUGCGUGACUGGUAAC GGUCAGGUGUGAAGCCCU CCCGACAAUGUAGCCCGA ACCGCAAGGUUGAAGCUG AAUCCGUGAGGAGGAAGC AACUUCACCAGUGUCAGG UGAUAGGGAACUAGGCUU GAGGGUAUGGUGAGCACA UGCGAAGUGAUGUCAGAA GCCUCGUCACAGACCAAC AGGCCAAAGACACUGAUA GGCCUGAGCCAAAACGGC AAAUGGAUAGGCUACAUC GCUCGCUCGUCGGUGUA CGGGGACGUCAAUCCAUC GGGGCACAGUCACCACCU AACCCCUCGUGUCAUCUG GUUGGAACGCGGUAAGCC CGUAUCCUCGCCUUGAAC ACUCAAGGCAGGCAAACC GUAAGGAAUGCUGAUGGG GGUGCGGGUAUGGGAUG CAGGAGAAAGCGAAUGCC GGUCUGUAAUGGACCGGA UAGGGGUUGAGGAGACAA UCCAACAUCACCCCGCCC GAAAGGGAGCAGACUUCC UGCUGGUCUCUCUUUGC GAGAUAGCCUGUAGAACC UCUUGAAUGGAGACAAGG CAAAUGGCAGUGGAACAA ACCACUGGUGCGGUCACC AACCAAACGGAAACAAGC UGGCACAGCAUAGACUGG GCCAAAGCCAACCGUGAG GUAAAGAGGCUGCAAGUG CGUAUCGCAAAGGCGUUC GCGCCGGUUCCUCUUGAA AGAGGGGCUUUGAGAGG CCUGAGCCGGAUGUGGG GAAACUCACAAGUCCGGU UCUUAGGGGGCGGGGAU GGCAGUAAUGCCUCCCUG CUACCCGGCGAUGACCUA

		CUCGAUUCAUGGAUCCUG UUG
